# Flavoromics Analysis of Passion Fruit-Roasted Chicken

**DOI:** 10.3390/foods13142221

**Published:** 2024-07-15

**Authors:** Ya Mao, Qi Liu, Jianwei Shao, Li Yang, Xuewu Zhang

**Affiliations:** College of Food Science and Engineering, South China University of Technology, Guangzhou 510640, China; miaya1999@163.com (Y.M.); liuqi312312@163.com (Q.L.); 202220126146@mail.scut.edu.cn (J.S.); feyangl@scut.edu.cn (L.Y.)

**Keywords:** passion fruit, roasted chicken, characteristic flavor components, flavor evolution pattern, flavoromics

## Abstract

Currently, research on the flavor components and their dynamic changes in roasted chicken with a special flavor is rare. In this study, a passion fruit-roasted chicken was prepared, its characteristic flavor components were profiled by flavoromics, and their evolution patterns and precursors were determined. The results showed that the characteristic flavor component with the highest contribution rate was ethyl butyrate (50.44%). In particular, some unique flavor compounds were identified compared with other roasted chicken products available. The main volatile flavor components in all stages of processing were alcohols, esters, and hydrocarbons, 15 to 30 min of roasting is an important stage for establishing the aroma system, and at the end, hydrocarbons were the main volatile compounds. During the 30-day storage period, the characteristic flavor components included ethyl butyrate, ethyl maltol, β-caryophyllene, and guaiacene. In conclusion, passion fruit-roasted chicken contained many characteristic flavor components, which were mainly formed within 15 to 30 min of roasting and were basically stable during the 30-day storage period. In a word, this work prepared a novel roasted chicken and revealed its mechanism of flavor formation at different baking stages and storage periods, which provided references for industrial production.

## 1. Introduction

The flavor of meat products is an important indicator and basis for consumers to choose products. Different types and proportions of flavor components constitute the aroma system of meat products, determining their unique aroma and affecting the quality of food [1]. The muscle protein tissue structure of chicken is tight, with abundant lipids distributed in the subcutaneous, intramuscular, and intramuscular regions. The types and content of unsaturated fatty acids in intramuscular fat are the most abundant, as well as various nutritional components such as free amino acids. These components undergo the Maillard reaction, lipid thermal degradation, and vitamin thermal decomposition under high-temperature reactions such as braising, roasting, deep frying, and high-pressure cooking [2,3,4,5]. In order to reveal the important flavor components of hot processed meat products at the molecular level, the key aroma factors that affect the product’s flavor need to be determined, to more accurately elucidate product flavor and provide a reference for strengthening and improving the characteristic flavor of the product.

Flavoromics technology is an effective method for targeted research on flavor components [6]. It analyzes the material basis of food flavor formation at the molecular level and conducts qualitative and quantitative analysis through technologies such as GC-MS, GC-IMS, and GC-O [7]. It can comprehensively describe the flavor characteristics of products and identify the main aroma-contributing components. Zhang et al. [8] analyzed the aroma components of melon wine using HS-SPME-GC-MS and LLE-GC-O-MS. They found that 3,6-nonadiene-1-ol and 3-nonadiene-1-ol were the reasons for the green and fatty taste of melon wine, while isoamyl acetate and (E)—Damascus ketone were the sources of its floral and fruity aroma. Ayseli et al. [9] identified the flavor characteristics of Türkiye chicken breast through the OAV method as a fresh leaf aroma and a cheese fat aroma. Baruth et al. [10] compared the volatile flavor compounds of wild pheasants and domestic chickens after roasting using SPME-GC-MS and GC-O. Among them, there were 66 species of wild pheasants, 62 species of mountain quails, 57 species of quails, and 45 species of domestic chickens; quantitative analysis showed that roasted domestic chickens had the highest characteristic aroma concentration, providing a reference for the development of pre-made poultry dishes.

Passion fruit has an elliptical or oval shape, its skin color is mostly purple or red, and the flesh is mainly yellow and orange, carrying a large amount of black seeds. Passion fruit is rich in characteristic aroma components such as ethyl butyrate, ethyl caproate, propyl acetate, 1-hexanol, and α-terpineol, which causes it to have a strong and attractive fruit aroma, hence neutralizing the greasiness of the meat; its bright color can also make the appearance of meat products more attractive and increase appetite [11]. On the other hand, passion fruit has the functions of clearing heat and removing wind, resolving phlegm, and stopping phlegm, and is mainly used to treat diseases such as wind heat, cold, rheumatism, dysmenorrhea, and insomnia [12]. Given the strong physiological activity and rich fruit aroma of passion fruit, it is often added as an aroma-enhancing ingredient to beverages and jams. At present, passion fruit juice is mainly used for beverage processing and jam processing, such as the production of compound juice drinks, vinegar, and compound fruit wine [13,14,15]. 

Traditional research on the flavor of chicken and its products at home and abroad is mostly focused on fresh chicken, braised chicken, smoked chicken, and salt-baked chicken. However, research on the flavor of roasted chicken with special flavor is rare, and research on the dynamic changes in aroma factors during the processing of roasted chicken with special flavor has not been reported. In addition, the analysis of flavor components and precursors of passion fruit-roasted chicken during processing and storage is good for optimizing the aroma composition and regulating odor generation. In our laboratory, a passion fruit-roasted chicken was prepared. The characteristic flavor components of the passion fruit-roasted chicken were profiled by employing flavoromics analysis, and the evolution patterns of flavor components during the processing and preservation were determined. Specifically, volatile flavor components in passion fruit-roasted chicken were first identified and the characteristic flavor components were determined; subsequently, the evolution patterns of various volatile flavor compounds and their precursors were analyzed, and their relationship in the processing was elucidated; finally, the changes in volatile flavor components during the preservation process were also investigated.

## 2. Results and Discussion

### 2.1. Analysis of Volatile Flavor Components in Passion Fruit Roasted Chicken

Using o-dichlorobenzene as the internal standard, the content of flavor components in passion fruit-roasted chicken was quantified. The specific types, contents, thresholds, EOAV values, and odor characteristics are summarized in Table S1. According to functional group classification, the main flavor substances in passion fruit-roasted chicken were aldehydes, ketones, alcohols, esters, hydrocarbons, ethers, and furans, with 7 categories and 44 volatile flavor substances in total. 

The generation of aldehydes mainly comes from the degradation and oxidative decomposition of chicken fat at high temperatures [16]. Six aldehydes were identified in passion fruit-roasted chicken, and the highest content was benzaldehyde (127.57 ng/g), followed by 3-furfural (83.65 ng/g) and then n-hexanal (57.21 ng/g). The pyrolysis products of amino acids participate in the Maillard reaction and Strecker degradation, thereby releasing small molecules of aldehydes. Benzaldehyde was a crucial aroma component generated by Strecker degradation of amino acids combined with lipid oxidation [17,18]. The open-chain form of sugars and the non-protonated form of amino groups have the highest reactivity in the Maillard reaction. When pH increases, it promotes carbonyl ammonia condensation. Therefore, it can be inferred that in the aroma system of passion fruit-roasted chicken, the source of hexanal is mainly the carbonyl ammonia reaction. Ketone substances can be generated by the re-oxidation of unsaturated fatty acids and the Maillard reaction [19]. Four types of ketone compounds were identified in passion fruit-roasted chicken, namely 5-methyl-2-hexanone (22.85 ± 2.76 ng/g), 3-hydroxy-2-butanone (58.91 ± 7.08 ng/g), 2-furanylacetone (14.13 ± 1.03 ng/g), and ethyl maltol (641.19 ± 38.50 ng/g). Among them, ethyl maltol is derived from the addition of pickles, which can effectively enhance food aroma. Seven types of alcohol substances were found, the representative substance of which is 2-methylbutanol (124.56 ng/g). Nine types of acids and esters were identified, among which the contents of ethyl butyrate and ethyl lactate are relatively high, with up to 149.22 ng/g and 137.36 ng/g, respectively. Ester compounds are generated by the reaction of alcohol intermediates from fat oxidation. The total flavor contribution rate of esters was 54.51%, among which ethyl butyrate had a contribution rate of up to 50.44%. This is the primary flavor substance of passion fruit-roasted chicken. Hydrocarbon compounds were the most diverse flavor compounds in passion fruit-roasted chicken. Among them, 15 types of hydrocarbon substances had the highest content, mainly including β-Caryophyllene (151.78 ng/g), toluene (50.63 ng/g), γ- Turpentene (46.17 ng/g), and limonene (31.50 ng/g). Furan compounds are important aromatic substances formed by the Maillard reaction of flavor precursors, which give meat products a rich meat aroma. The furan compound identified in passion fruit-roasted chicken was 2-pentylfuran, with a content of 5.67 ng/g, which is a common flavor substance in meat products. Its main source is aldehyde oxidation [20]. 

To further explore the main aroma-contributing components in passion fruit-roasted chicken, it was necessary to combine the content of various flavor components and their thresholds to make judgments. When EOAV > 1, it was determined as the main flavor component. According to Table S1, 16 flavor components had an EOAV value greater than 1, including 5 aldehydes, 1 ketone, 2 alcohols, 3 esters, 3 hydrocarbons, and 2 ethers. Nonanal had a low odor threshold value and was related to lipid oxidation with green and fatty description [21]. N-hexanal, L-linalool, and phenylethanol β-Caryophyllene contributed to the fragrance of flowers such as grass, pine, gardenia, and lily of the valley [22]. 3-Furfural and ethyl maltol were identified as the main aroma-contributing components, playing an important role in forming the sweet caramel and roasted taste of passion fruit-roasted chicken, and it also had a certain bread aroma [23]. Anisin and anethole had a sweet anise aroma and licorice flavor, giving them a certain spice flavor. Guaiacol had a smoky odor characteristic and was a volatile component that constitutes the smoky aroma of passion fruit-roasted chicken [24].

To obtain characteristic flavor components, it was necessary to more intuitively characterize the contribution rate of flavor components to the overall flavor, which is known as the odor contribution rate [25]. As shown in Table S1, the flavor component with the highest contribution rate in the passion fruit-roasted chicken was ethyl butyrate (50.44%), while the other flavor components with a contribution rate greater than 3% were isovaleraldehyde (3.15%), n-hexanal (3.87%), 3-furfural (3.53%), guaiacene (8.77%), phenylethanol (14.81%), and ethyl maltol (4.93%), respectively, with flavor components greater than 3% accounting for 89.50% of the total contribution rate, basically representing the overall flavor characteristics of passion fruit-roasted chicken. It is clear that there are seven characteristic flavor components of passion fruit-roasted chicken, namely isovaleraldehyde, hexanal, 3-furfural, ethyl maltol, phenylethanol, ethyl butyrate, and guaiacene. The main aroma characteristics of passion fruit-roasted chicken are sour and sweet fruit aroma, grass aroma, floral and woody aroma, caramel flavor, and smoky flavor, supplemented by spices such as fennel and licorice.

### 2.2. The Time-Course Changes in Volatile Flavor Components during the Processing of Passion Fruit-Roasted Chicken

The types and content changes in volatile flavor components obtained HS-SPME-GC-MS in the preparation and roasting process of passion fruit-roasted chicken are shown in Table S2. At different processing stages, a total of 89 volatile flavor components were identified. The types and content of volatile flavor components show an overall trend of increasing first and then decreasing. The number of types of volatile flavor components in raw chicken was the lowest, with 25 types and 452.32 ng/g in total. Only hexanal (240.86 ng/g) (a characteristic volatile flavor component), glutaraldehyde (11.23 ng/g), and nonanal (6.35 ng/g) (two main volatile flavor components) were detected. After 15 min of roasting, all seven characteristic volatile flavor components were detected, and the highest number of volatile flavor components was detected at 60 min. The content of volatile flavor components also rapidly increased to 1502.68 ng/g (*p* < 0.05), significantly higher than that of raw meat and pickled samples (741.46 ng/g). After being roasted for 30 min, 45 min, 60 min, and 70 min, all seven characteristic volatile flavor components were detected. At 30 min, the total volatile flavor components during the roasting process reached their maximum content of 4020.17 ng/g, which is significantly different from other stages (*p* < 0.05). It can be concluded that the key aroma stage of passion fruit-roasted chicken occurs at 15–30 min of roasting, during which a large number of volatile flavor components from chicken and spices are generated and released. The characteristic volatile flavor components, except for isovaleraldehyde, reached their maximum values, which is an important stage in the formation of the aroma system of passion fruit-roasted chicken. With the extension of roasting time, after 45 min of roasting, the total number of volatile flavor components gradually decreased and tended to stabilize. At 45 min, 53 compounds were detected, accounting for 2816.60 ng/g in total. The high temperature further volatilizes the volatile flavor components, which decrease to 2119.28 ng/g after 60 min of roasting. Generally speaking, many flavor components were present in passion fruit-roasted chicken but absent in raw roasted chicken without passion fruit (Table S2), which is dynamic during processing. These include aldehydes (isovaleraldehyde, 3-furfural, benzaldehyde, and phenylacetaldehyde), ketones (3-hydroxy-2-butanone, ethyl maltol), alcohols (2-methylbutanol, linalool oxide, L-Linalool, terpineol, and Phenylethanol), acids and esters (ethyl acetate, ethyl butyrat, ethyl caproate, ethyl lactate, 1-Methylbutyrate hexyl ester, hexyl butyrate, ethyl octanoate, and ethyl 3-hydroxybutyrate), hydrocarbons (3-carene, β-pinene, Guaiacol, and β-caryophyllene), and ethers (dimethyl ether, anethole). In order to ensure that the volatile flavor components can withstand high temperatures and be stored in the finished product, a re-drying process was carried out after 60 min. The total amount of volatile flavor components in the final passion fruit-roasted chicken product was 2844.18 ng/g.

### 2.3. Evolution Patterns of Various Volatile Flavor Compounds

The types and proportions of flavor components indicated that alcohols, esters, and hydrocarbons were the main volatile flavor components in all stages of processing (Table S2). With the extension of roasting time, aldehydes generated by the transformation of various compounds become the main flavor components. At the end of the roasting stage, various compounds gradually evaporated at a high temperature of 200 °C, and ultimately, hydrocarbon substances were the main volatile compounds in passion fruit-roasted chicken.

**Figure 1:**
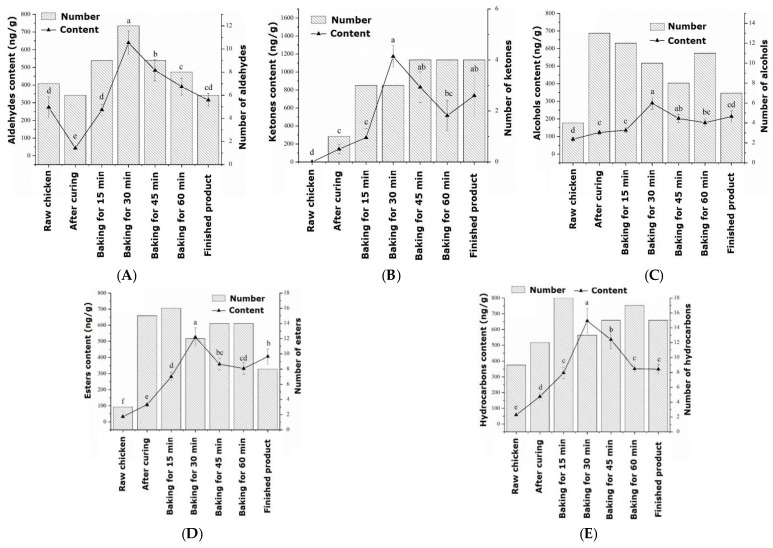
Changes in volatile flavor compounds during the processing of passion fruit-roasted chicken: (**A**) aldehydes, (**B**) ketones, (**C**) alcohols, (**D**) esters, and (**E**) hydrocarbons. a–f: different letters indicate statistical significance (*p* < 0.05) between different groups.

The changes in aldehyde substances during the processing of passion fruit-roasted chicken are shown in Figure 1a. During the entire roasting process, a total of 16 aldehydes were identified, which first increased and then decreased during processing and reached their maximum values after 30 min of roasting. In raw chicken, the flavor of aldehydes accounts for the highest proportion at 60.55%, which is the compound with the highest proportion in raw meat, mainly derived from hexanal. The aldehydes that could be detected throughout the entire process from raw chicken to cooked products were glutaraldehyde, hexanal, and nonanal. When heating began, the identified aldehydes were isovaleraldehyde, 3-furfural, and phenylacetaldehyde. As the roasting process progressed, the content of aldehydes significantly increased (*p* < 0.05), with a total amount of 638.82 ng/g at 30 min. There were still many retained aldehydes in the finished roasted chicken, including 3-furfural and benzaldehyde, with values of 83.65 ng/g and 127.57 ng/g, respectively. Within 30–45 min of roasting, a small amount of heptanal is generated, which is believed to cause a fatty and rancid odor to meat products [26]. 

The changes in ketone substances during the processing of passion fruit-roasted chicken are shown in Figure 1b. During the entire roasting process, a total of seven types of ketone compounds were detected, and by 45 min of roasting, there were four types of ketone compounds with the highest flavor proportion (29.50%). The main source was ethyl maltol, which was released into the meat in large quantities at high temperatures, producing a strong sweet caramel flavor. In raw chicken, no ketone substances were detected, and 5-methyl-2-hexanone was only detected after the high temperature of 200 °C.

The changes in alcohol substances during the processing of passion fruit-roasted chicken are shown in Figure 1c. During the entire roasting process, a total of 16 types of alcohol substances were detected, similar to the changes in aldehydes, which first increased and then decreased, reaching a maximum content of 289.22 ng/g after 30 min of roasting. In raw chicken, only four types of alcohols were detected, namely pentanol, hexanol, 3-octanol, and heptanol. Pentanol mainly resulted from the degradation of lipid hydroperoxides, and hexanol was derived from linoleic acid [27]. In the cured chicken, 13 types of alcohol substances were detected, indicating that some volatile alcohol substances in the pickling materials and passion fruit powder were released during the pickling process. The content of the important flavor substance L-linalool was significantly higher in the early stage of roasting than in the pickling stage (*p* < 0.05), reaching a maximum content of 29.44 ng/g at 30 min of roasting, and eventually decreasing to 16.24 ng/g. The alcohol that could be detected throughout the entire process was hexanol, which has a fruity and sweet aroma. 

The changes in ester substances during the processing of passion fruit-roasted chicken are shown in Figure 1d. During the entire process, 18 esters were identified, which increased first, then stabilized, and finally decreased. When baked for 30 min, the total content reached its maximum value of 525.95 ng/g, significantly higher than the ester content at 15 min (280.18 ng/g). Additionally, two types of acidic substances were identified, which may be oxidation products of alcohols and aldehydes and hydrolysis products of triglycerides and phospholipids, but their volatility was low and they had no significant effect on flavor [28].

The changes in hydrocarbon substances during the processing of passion fruit-roasted chicken are shown in Figure 1e. Hydrocarbons are the most diverse compounds identified during the roasting process, with a total of 27 types, which reached 654.77 ng/g with a rapid increase before 30 min of roasting. The increase in caryophyllene was the largest, increasing from 6.68 ng/g and 126.29 ng/g after pickling to 50.95 ng/g and 409.07 ng/g after roasting for 30 min, respectively. 

During the entire roasting process, two types of other substances were detected, namely methyl ether and anethole, which made the meat aroma of chicken more prominent and had a significant synergistic effect, bringing a pleasant and mellow feeling [29]. Only one heterocyclic compound, 2-pentylfuran, was identified, which plays an important role in the flavor system of meat products due to its meaty and buttery aroma. Its content was relatively low in the early stages of roasting, gradually increasing with the oxidation of fat up to 7.29 ng/g after 30 min of roasting and 6.84 ng/g after 45 min. However, it was inhibited by spices and was not detected after 60 min of roasting. 

### 2.4. Evolution Patterns of Flavor Components

**Figure 2:**
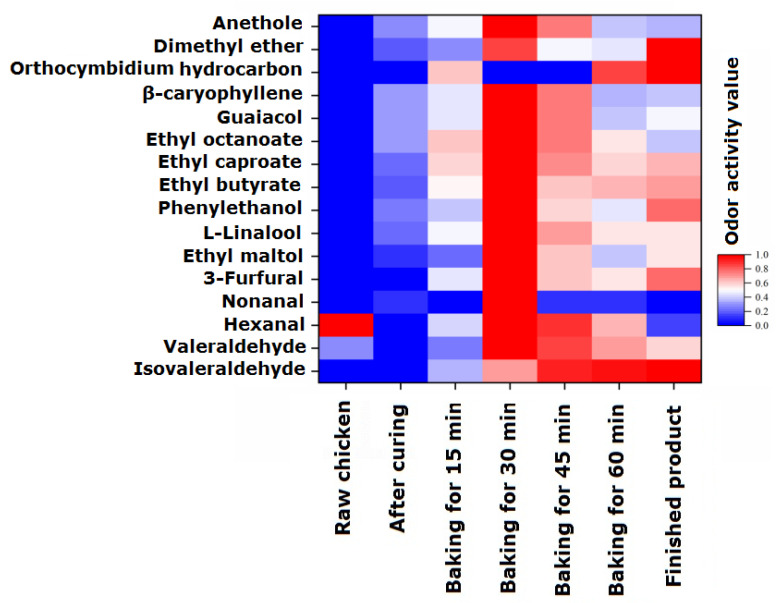
Changes in the estimated odor activity values (EOAVs) of characteristic flavor components during the processing of passion fruit-roasted chicken. The EOAVs of characteristic flavor components were normalized (0–1).

To analyze the odor activity changes in 16 volatile flavor components with EOAV > 1 in passion fruit-roasted chicken during the processing and roasting process, they were normalized and the results are shown in Figure 2. In raw chicken, only three flavor substances, valeraldehyde, hexanal, and nonanal, had EOAV > 1, and their EOAV values were 1.40, 48.17, and 1.49, respectively, which is consistent with the research conclusions of Wang et al. [30]. Hexal is the most important aldehyde substance in Wenchang chicken, accounting for approximately 85% of the aldehyde substances, followed by valeraldehyde and nonanal. In the marinated chicken samples, there were 11 substances with EOAV values > 1, which come from spices that have not yet undergone heating reactions. When the heating time was prolonged, the overall EOAV values of the seven characteristic volatile flavor components gradually increased, reaching their maximum values after 30 min of roasting, which is basically consistent with the changes in the content of volatile flavor components. The EOAV values slowly decreased during the 30–60 min roasting stage. At 60 min, the flavor compounds with higher EOAV values were ethyl butyrate, hexanal, phenylethanol, and guaiacene. Moreover, the EOAV value of ethyl butyrate was greater than 100 after 15 min of heating, indicating that heating causes the continuous release of a large amount of ethyl butyrate with pineapple and saffron fragrance, which adhere to the chicken tissue and causes the chicken to emit a fresh fruity aroma. The re-drying process slightly increased the EOAV of the sample after 70 min, with the highest increase of 74.1% in the EOAV value of phenylethanol, contributing to the fragrance of lilac flowers and also playing a role in fragrance retention.

### 2.5. Evolution Patterns for Precursors of Flavor Substances

The main components (protein and fat) and their content changes in passion fruit-roasted chicken are shown in Table S3. In addition to their physiological functions, proteins and fats are important precursors for the production of volatile aroma compounds in chicken. Proteins can undergo complex chemical reactions with carbonyl-containing substances such as reducing sugars, such as the Maillard reaction, while generating a large number of small-molecule volatile compounds [31]. During the entire period of processing and roasting, the protein content ranged from 52.32% to 70.77%. Fat can affect the tenderness and juiciness of meat products and also act as a precursor substance to affect the flavor and taste of meat products, producing different aromas during different heating processes [32]. Fat hydrolysis can produce fatty acids, while unsaturated double-bond oxidation can produce peroxides that can produce volatile substances such as aldehydes, ketones, alcohols, carboxylic acids, etc. The fat content changed in the range of 11.85% to 19.36%, with raw chicken having the highest fat content. After heating for 15 min, the fat content dropped to 11.85%, showing a significant change (*p* < 0.05). 

#### 2.5.1. Free Amino Acids and Their Contents during the Processing

**Figure 3:**
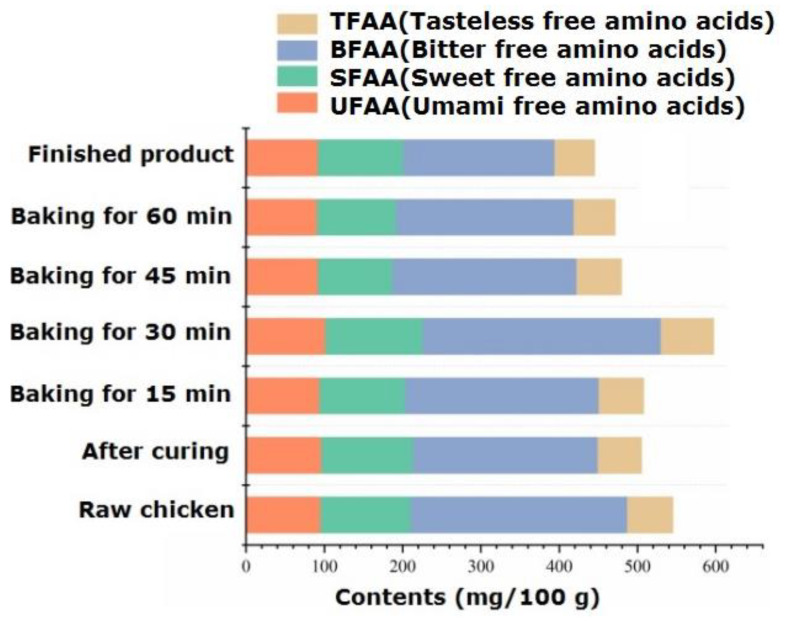
Dynamic changes in flavor amino acids during the processing of passion fruit-roasted chicken.

The types and variation patterns of free amino acids in passion fruit-roasted chicken are shown in Table S4. Free amino acids, as flavor precursors, play an important role in aroma and taste. During the processing, a total of 17 free amino acid components were identified, which significantly decreased from 545.25 mg/100 g in raw chicken to 505.19 mg/100 g in pickled chicken. The possible reason is that soaking in salt water dissolved many amino acids [33]. Free amino acids not only serve as precursors of volatile aroma components but also enhance the taste of meat products. Li et al. [34] found that the free amino acids that contribute significantly to the taste of chicken are alanine, glutamic acid, and arginine. From the perspective of flavor characteristics, amino acids are divided into UFAA (umami free amino acids: glutamic acid and aspartic acid), SFAA (sweet free amino acids: alanine, glycine, serine, and threonine), BFAA (bitter free amino acids: methionine, isoleucine, leucine, arginine, valine, tyrosine, phenylalanine, and histidine), and TFAA (tasteless free amino acids: lysine, proline, and cysteine). Figure 3 shows the changes in the content of amino acids with different flavors during processing. For raw chicken, the top three amino acids regarding content were glutamic acid, histidine, and arginine, with contents of 63.46 mg/100 g, 58.41 mg/100 g, and 57.74 mg/100 g, respectively. Among them, glutamic acid is an important UFAA, and its content significantly decreased (*p* < 0.05) after 60 min of roasting, dropping to 56.50 mg/100 g. The content of SFAA significantly increased during 30 min of roasting, with serine being more prominent in the finished product, giving it a sweet and refreshing taste. In the finished product, BFAA, SFAA, and UFAA accounted for 43.42%, 24.48%, and 20.72% of the total amino acids, respectively, similar to the distribution of flavored amino acids in black Angus beef [35]. The content comparison shows the order of BFAA > SFAA > UFAA, among which BFAA has the effect of reducing blood pressure and fat in the diet [36].

#### 2.5.2. Flavor Nucleotide Composition and Content

The types of flavor nucleotides and their degradation products in passion fruit-roasted chicken determined by HPLC are shown in Table S5. In meat products, inosine monophosphate (5′—IMP), guanosine monophosphate (5′—AMP), and adenosine monophosphate (5′—AMP) are the main flavor nucleotides, which are produced by the degradation of adenosine triphosphate (ATP). In meat products, the main degradation pathway of ATP is as follows: adenosine triphosphate (5′—ATP) continuously removes the phosphate group twice to form adenosine monophosphate (5′—AMP), adenosine monophosphate removes the amino group to form inosine monophosphate (5′—IMP), inosine acid is degraded to inosine (I) by removing phosphate groups, and inosine is degraded to hypoxanthine (Hx) by removing bases.

In raw chicken, Hx and I nucleotides are the main components with higher content, which may be produced by refrigerated meat products. After pickling, the content of 5′—IMP and 5′—GMP sharply decreased (*p* < 0.05), with a total amount of flavor nucleotides of only 11.71 mg/100 g. This is because organic acids such as acetic acid and hexanoic acid are produced during the pickling process, and the acidic environment causes the degradation of flavor nucleotides [37]. At the beginning of heating, the content of 5′—AMP and 5′—IMP significantly increased, being 11.5 times and 6.42 times higher than the post-pickling values, respectively. After heating for 60 min, the content of 5′—IMP increased to 77.09 mg/100 g, which was the highest point in the entire process. 

In meat products, flavor nucleotides have a significant impact on taste and flavor, but sometimes their content levels are lower. Xiong et al. [38] reported that chicken contains abundant compounds of 5′—nucleotides. Chen et al. [39] indicated that the main nucleotide component of crab meat is AMP, followed by IMP, with a relatively low GMP content (2.3 mg/100 g). Consistent with the research findings of Liu et al. [40], the concentrations of nucleotide ADP, AMP, and GMP in duck meat are relatively low. However, nucleotides play an important role in flavor multiplication in meat products. ATP and its degradation products have a synergistic effect with fresh peptides, free amino acids, organic acids, etc., enhancing freshness while promoting the production of volatile flavor compounds, releasing the unique aroma of the passion fruit and chicken fusion.

#### 2.5.3. Free Fatty Acid Components and Content

The fat in animal meat has a crucial impact on the production and processing of characteristic flavor meat. The types and contents of fatty acids in the processing of passion fruit-roasted chicken are shown in Table S6. The intramuscular fat of chicken is composed of phospholipids, triglycerides, and free fatty acids, among which free fatty acids are important nutritional factors, flavor substances, and flavor precursors. Unsaturated fatty acids are prone to lipid peroxidation due to their presence of unsaturated bonds. The resulting peroxides are degraded to produce small-molecule flavor substances such as aldehydes, ketones, alcohols, carboxylic acids, etc. Therefore, unsaturated fatty acids play an important role in the formation and improvement of the flavor of passion fruit-roasted chicken. The highest content of fatty acids was found in raw chicken, which was 5874.59 mg/100 g. With heating, the total amount of fatty acids significantly decreased (*p* < 0.05), reaching its lowest point during the 15–30 min roasting stage and fluctuating within the range of 3375.85~3398.19 mg/100 g.

**Figure 4:**
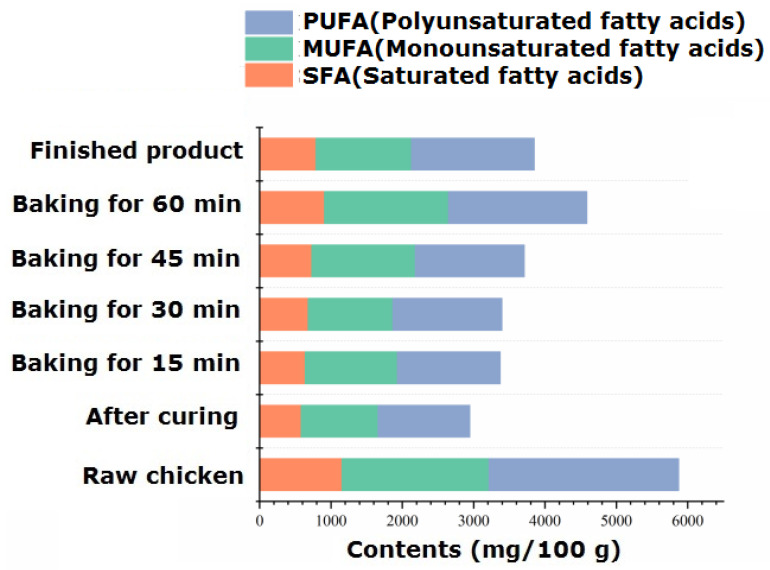
Dynamic changes in different kinds of fatty acids during the process of passion fruit-roasted chicken.

According to Table S6 and Figure 4, a total of 34 free fatty acids were identified, including 13 SFAs, 9 MUFAs, and 12 PUFAs. During the processing and roasting process, the unit content of the three fatty acids was PUFA > MUFA > SFA, and the proportion of PUFA to the total fatty acids varied between 41.18% and 45.25%. Palmitoleic acid (C16:1) and oleic acid (C18:1) were the main monounsaturated fatty acids; linoleic acid (C18:2), linolenic acid (C18:3), arachidonic acid (C20:4), and docosadienoic acid (C22:2) were the primary polyunsaturated fatty acids, and their content significantly decreased after 70 min of roasting (*p* < 0.05). Research has confirmed that polyunsaturated fatty acids such as linoleic acid can undergo pyrolysis to produce acrolein [41], while monounsaturated acids such as oleic acid can undergo pyrolysis to produce substances with carboxylic acid groups. A research group once studied the reaction model of the effect of free fatty acids on pork flavor and found that oxidized pork fat can participate in the Maillard reaction to produce aromatic compounds [42]. The alcohols and carboxylic acids produced by the degradation of unsaturated fatty acids in chicken can also self-cyclize to form meat-flavored lactone compounds [43]. They can also undergo esterification reactions with the alcohols and carboxylic acids rich in additives, especially passion fruit, to increase the content of ester compounds and make the fruit aroma more mellow and rich.

#### 2.5.4. Soluble Sugar Components and Content

Carbohydrates not only provide energy substrates, but also undergo hydrolysis reactions, Maillard reactions, and caramelization reactions under conditions such as heating, enzymes, and amino acids, generating monosaccharides, flavor compounds, and polymeric pigments. They are important nutritional sources, flavor components, and precursor substances in chicken processing. The specific types and content changes regarding soluble sugars are shown in Table S7. The total soluble sugar content in raw chicken is 41.91 mg/100 g, which is the lowest stage of sugar content during the processing. Only glucose and ribose were detected, with contents of 29.30 and 12.23 mg/100 g, respectively, consistent with Liu et al. [44]. At the beginning of the heating process, the total amount of soluble sugars significantly decreased (*p* < 0.05) after 15, 30, and 45 min of roasting, reaching 93.81, 80.33, and 72.80 mg/g, respectively. From the perspective of the types of soluble sugars, a total of nine sugars were identified throughout the processing, mainly glucose, fructose, and maltose, accounting for approximately 95% of the total soluble sugars. It can be seen that pickles and passion fruit powder provide important flavor precursors for the product. The reducing sugars contained in them can react with the amino components in chicken via the Maillard reaction. Different types and ratios of soluble sugars bring unique flavors to chicken. 

### 2.6. Correlation Analysis between Characteristic Flavor Components and Flavor Precursors

**Figure 5:**
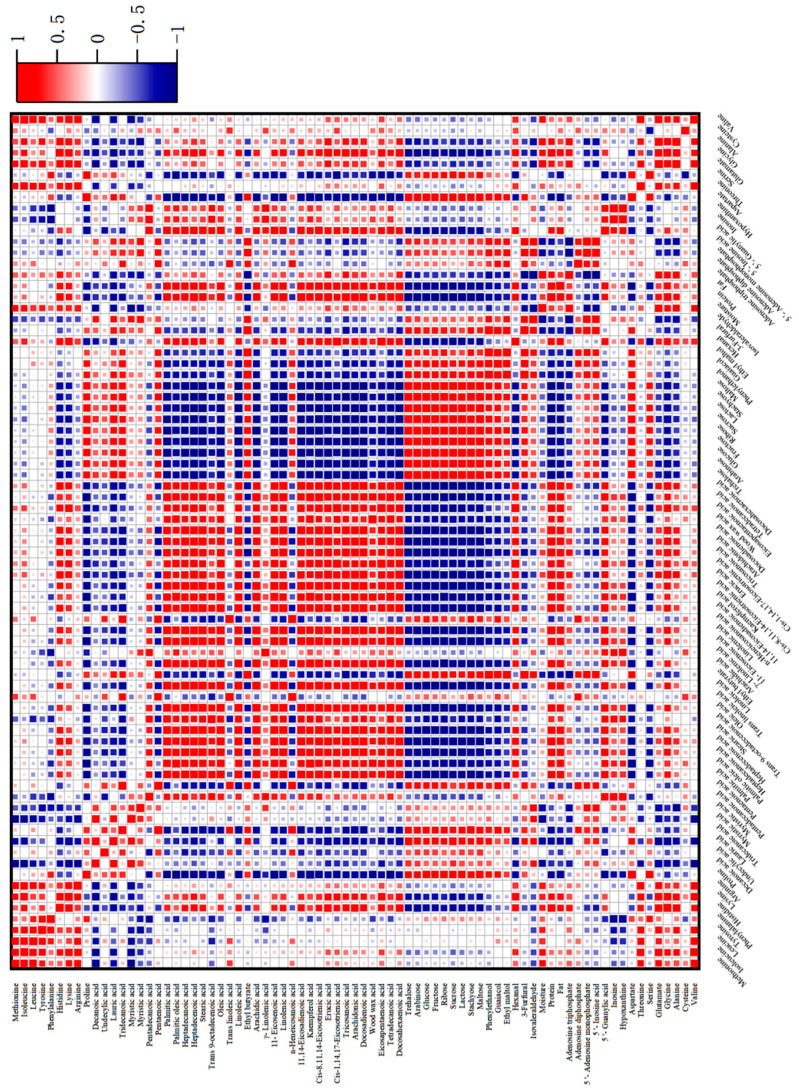
Correlation between characteristic volatile components and flavor precursor substances in passion fruit-roasted chicken. Figure 5 was produced by OriginPro 2022.

The correlation between volatile flavor components and flavor precursor substances during the processing is shown in Figure 5. The negative correlation between ethyl butyrate and the basic nutrients of water (r = −0.67**) and protein (r = −0.55*) was high. In particular, ethyl butyrate was negatively correlated with fatty acid compounds, such as twenty-three acid (r = −0.69**), oleic acid (r = −0.44*), linoleic acid (r = −0.48*), linolenic acid (r = 0.50*), and arachidonic acid (r = −0.48*), indicating that fatty acids are important substrates affecting the release of aroma from ethyl butyrate. Hexanal was negatively correlated with three free amino acids: proline (r = −0.59*), serine (r = −0.64**), and aspartic acid (r = −0.35*). It was also negatively correlated with fatty acids such as decanoic acid (r = −0.63**), lauric acid (r = −0.66**), and myristic acid (r = −0.35*). There was a significant negative correlation with nine sugars, with sucrose (r = −0.81**), lactose (r = −0.80**), and maltose (r = −0.63**) showing the strongest correlations. The open-chain form of sugars and the non-protonated form of amino groups exhibit the highest reactivity in the Maillard reaction, and an increase in pH promotes carbonyl ammonia condensation [45]. 

There were many types of free amino acids negatively correlated with isovaleraldehyde, with a total of 14 types. This may be due to the involvement of the pyrolysis products of amino acids in the Maillard reaction and Strecker degradation, thereby releasing small molecules of aldehydes [46]. However, most fatty acids were positively correlated or not significantly correlated with isovaleraldehyde, and soluble sugars also had similar patterns. The correlation between phenylethanol, guaiacene, and 3-furfural with flavor precursor substances followed a similar pattern. They had a significant negative correlation with long-chain fatty acids, especially palmitic acid, oleic acid, linoleic acid, arachidic acid, 11-eicosylic acid, linolenic acid, lignic acid, and docosahexadilute acid, indicating that the oxidation reaction of fatty acids may be an important substrate source. It is also possible that the oxidation products of long-chain fatty acids have a promotional effect on the synthesis of these three substances. From the heat map (Figure 5), it can also be seen that the three had a significant negative correlation with adenosine triphosphate (5′—ATP), with correlation coefficients of r = −0.78**, r = −0.63**, and r = −0.89**, respectively. Furthermore, there was a significant positive correlation between adenosine diphosphate (5′—ADP) and adenosine monophosphate (5′—AMP), which may be due to the synergistic effect between the degradation of 5′—ATP and the generation of characteristic flavor components, while the early degradation products of 5′—ATP had an antagonistic effect on the generation of aroma components.

### 2.7. Changes in Volatile Flavor Components during Preservation Process

The flavor component changes in the roasted chicken made from passion fruit after vacuum compression and other packaging within 30 days are shown in Table S8. During the 30-day storage period, a total of 70 volatile flavor components were identified, which was 21.34% less than the number of types identified during the processing. The types and content of volatile flavor components showed a pattern of first increasing and then decreasing. Studies have reported that the types and contents of aldehydes, ketones, hydrocarbons, and heterocyclic compounds in preserved duck jerky continue to decrease [47]. After 10 days of storage, the total amount of volatile flavor components decreased from 2844.18 ng/g to 2163.87 ng/g at the end of roasting, indicating a high degree of aroma retention. After 15 days, the total amount significantly increased (*p* < 0.05), and flavor precursor substances decomposed and transformed into volatile small-molecule substances. After 20 days, the main flavor substances were β-Caryophyllene and ethanol, which accounted for 33.26% of the total amount. After 30 days of storage, there were only 31 volatile substances, with the highest proportions of alcohols (618.49 ng/g) and hydrocarbons (632.16 ng/g) of up to 35.46% and 37.0%, respectively. During the storage period, no amine substances such as aniline, which can spoil the flavor, were detected. From the classification of functional groups of volatile aroma compounds, it can be seen that during the storage period, the volatile flavor components were still mainly aldehydes, ketones, alcohols, esters, and hydrocarbons, with a high degree of retention of various compounds. Among them, hydrocarbon substances were the primary flavor components during the storage period, consistent with the processing and roasting stages. The analysis of flavor components for different classifications is described next. 

A total of 10 types of aldehydes were identified, and there were 7 types of aldehydes in the first 10 days. As the storage period extended, the types of aldehydes gradually decreased, and after 15 days, they significantly decreased. On the 30th day, there were only three types of aldehydes (benzaldehyde, nonanal, and nutmeg aldehyde), and the content of aldehydes was only 29.80% of the highest value during the storage period (731.28 ng/g). Nonanal always exists during the processing and storage period, which, along with other small components, forms the characteristic flavor of passion fruit-roasted chicken. Seven types of ketones were identified during the storage period, and their types and contents significantly decreased after 20 days (*p* < 0.05). After 30 days, only two types of ketones were present. Among them, the preservation effect of ethyl maltol was the most obvious, and the content gradually decreased within the range of 53.66~280.57 ng/g within 30 days, which is related to the fragrance-holding and shelf-life-extension functions. Dihydrogen- β- Violet ketone was present throughout the entire preservation process, and when mixed with ethanol, it can produce a violet aroma.

During the preservation period, seven types of alcohol substances were identified, including L-linalool, oxidized linalool, and α- Terpineol, and three other alcohols were retained to a high extent. The overall content of L-linalool ranged from 16.14 to 45.72 ng/g, retaining the woody aroma of passion fruit-roasted chicken. The content of α- turpentine alcohol was 20.88 ng/g after 25 days, and there was no significant change during the storage period. Ethanol was the primary alcohol substance during the storage period, accounting for more than 70% of the total alcohol content, and its content significantly increased with the extension of shelf life (*p* < 0.05). This may be due to the conversion of glucose in chicken under anaerobic conditions, as well as the generation of small-molecule volatile substances such as aldehydes, ketones, acids, and esters, bringing a sweet and mellow taste. However, excessive ethanol can also produce a rancid taste. Lastly, there were 14 types of ester substances identified. The total amount of ester compounds significantly increased after the 15th day (*p* < 0.05) to 393.03 ng/g, mainly derived from 3-heptanyl hexanoate, which is a common ester flavor. The volatile flavor component, ethyl butyrate, always exists during the storage period (15.39~70.84 ng/g), and the EOAV value was greater than 15 throughout the storage period. Hydrocarbon compounds were the most diverse compounds identified during the preservation period, with a total of 29 types. Within 25 days, there was little change in the types of hydrocarbon substances, but after 30 days, the types sharply decreased, with only 12 types. β- Caryophyllene and its isomers are still important aroma-contributing components in hydrocarbon compounds, with EOAV values greater than 1 during the storage period. 

## 3. Conclusions

Flavoromics technology is an effective method for research on flavor components. Using this technology, the top contribution rate of flavor components to the overall flavor of passion fruit-roasted chicken was determined as ethyl butyrate (50.44%), phenylethanol (14.81%), guaiacene (8.77%), ethyl maltol (4.93%), n-hexanal (3.87%), 3-furfural (3.53%), and isovaleraldehyde (3.15%). During the roasting process, these characteristic flavor components were dynamic: they first increased, reaching their maximum values at 30 min of roasting, then slowly decreased during the 30–60 min roasting stage. During the preservation process, the types and content of flavor components showed a pattern of first increasing and then decreasing. After 30 days of storage, there were only 31 volatile substances, with the highest proportions of substances being alcohols (35.46%) and hydrocarbons (37%). 

On the other hand, during the roasting process, the main precursors of flavor components evolved in different patterns. The protein content was high (70.77%) in raw chicken, and decreased in the roasting period, but was basically maintained at the level of 53.35%-56.48%. Fat content was also high (19.36%) in raw chicken and decreased in the roasting period, but first increased from 11.85% to 17.82% before 45 min of roasting and then dropped to 12.23% in the finished product. In contrast, the content of soluble saccharides was low (41.91 mg/100 g) in raw chicken but markedly increased during the roasting period, being maintained at the level 7279.62 mg/100 g–9381.19 mg/100 g. 

Finally, it is noted that a number of flavor components were present in passion fruit-roasted chicken but absent in raw roasted chicken without passion fruit (Table S2), with the main components including aldehydes (isovaleraldehyde, 3-furfural, phenylacetaldehyde), ketones (3-hydroxy-2-butanone), alcohols (linalool oxide), acids and esters (ethyl acetate), and hydrocarbons (β-pinene). In particular, some flavor components were present in passion fruit-roasted chicken but absent in other pre-made chicken products (Table S9), such as ketones (5-methyl-2-hexanone, 3-hydroxy-2-butanone, and 2-furanylacetone), alcohols (2-methylbutanol, 2-Heptanol, Hexanol, (S)—Linalool oxide, L-Linalool, Terpineol, and Phenylethanol), acids and esters (ethyl acetate, Ethyl Butyrate, ethyl lactate, 1-Methylhexyl ester, Hexyl butyrate, Ethyl octanoate, and ethyl 3-hydroxybutyrate), hydrocarbons (β-myrcene, (+)—limonene, ocimene, styrene, Guaiacol, β-caryophyllene, 1-Pentene, and Anisin), ethers (dimethyl ether), and phenols (ethyl maltol). In summary, passion fruit-roasted chicken is a novel product with unique flavor components. 

## 4. Materials and Methods

### 4.1. Materials

Wenchang chicken was purchased from Wenchang City, Hainan Province, China. Passion fruit was sourced from the planting base in Haikou, Hainan Province, China. Composite phosphates, ethyl maltol, isomalto oligosaccharides, and pectinase (20,000 U/g) were all food grade and purchased from Wanxiang Hongrun Biotechnology Co., Ltd., Chengdu, China. Adenosine triphosphate (5′—ATP), adenosine diphosphate (5′—ADP), 5′-adenosine monophosphate (5′—AMP), 5′—guanosine monophosphate (5′—GMP), 5′—inosine monophosphate (5′—IMP), hypoxanthine (Hx), and inosine (I) were all of chromatographical purity (>98%) and purchased from Yuanye Biotechnology Co., Ltd., Shanghai, China. Free amino acids, soluble sugars, and fatty acid standards were all analytical grade and purchased from Sigma Aldrich (St. Louis, MO, USA).

### 4.2. Pickling and Roasting of Chicken

Wenchang chicken leg (weight 100 ± 30 g) was selected, followed by the removal of fat, fascia, and congestion, cleaning, and the draining of water at room temperature. External pickling solution (5% Anchor butter (Dairy Brands Limited Co., Christchurch, New Zealand), 5% honey (Guanshengyuan Limited Co., Guangzhou, China), 5% yellow wine (Hengshun Limited Co., Zhenjiang, China), 0.6% light soy sauce (Haitian Limited Co., Foshan, China), 0.4% black pepper (Weihaomei Limited Co., Guangzhou, China), and 0.4% table salt (Haiyan Limited Co., Guangzhou, China)) were applied on the surface of the chicken. The internal pickling solution (composite phosphate 0.36%, ethyl maltol 0.02%, oligomeric isomaltose 0.85%, and passion fruit powder 16.55%, prepared by pulping, homogenizing and spray-drying) was purchased from Chengdu Vientiane Hongrun Biotechnology Co., Ltd., Chengdu, China and evenly injected into the chicken using a 5 mL syringe, packaged into a pickling bag, sealed with cling film in the container, and refrigerated at 4 °C for 10 h. Then, the marinated chicken was roasted in a rotary oven with gradient heating: the first stage was 120 °C, lasting for 60 min, and the direction was adjusted at 30 min; 60 min later, the roasted chicken was removed and vented in the air by puncturing. The marinated solution (3% butter, 3% honey, 2.5% yellow wine) was evenly applied on the surface of the chicken, repeating the process three times. In the second stage, the chicken was put back into the oven for re-drying at a temperature of 200 °C for 9 min, and a bright orange–yellow roasted chicken with the characteristics of passion fruit was obtained. During the processing, samples were taken from seven process points: raw chicken, marinated chicken, chicken roasted for 15 min, chicken roasted for 30 min, chicken roasted for 45 min, chicken roasted for 60 min, and the finished product. Using extraction needles to adsorb volatile aroma compounds from samples and using gas chromatography-mass spectrometry detection, the dynamic changes in volatile flavor components during the processing and roasting of passion fruit-roasted chicken were qualitatively and quantitatively analyzed.

### 4.3. HS-SPME-GC-MS (Headspace Solid-Phase Microextraction Gas Chromatography Mass Spectrometry) Analysis

Headspace solid-phase microextraction (SPME): SPME needles (50/30 μm DVB/CAR/PDMS from Supelco, Bellefonte, PA, USA) were used, 5.0 g of the roasted sample was transferred immediately to an automatic headspace injection bottle, internal standards were added, the bottle was quickly sealed with polytetrafluoroethylene spacers, and the sample was mixed thoroughly. The SPME extraction needle was first activated at 280 °C for 30–60 min and then activated at 270 °C for 15 min each time. The analysis time was 5 min. The headspace injection bottle was preheated in a water bath at 45 °C and 250 r/min for 20 min, and the activated SPME extraction needle was used for 40 min at 45 °C. Then, GC-MS was performed. The inlet temperature was 250 °C. Desorption time was 5 min. The heating process consisted of four gradients: 45 °C for 3 min; rising to 180 °C at a speed of 6 °C/min; rising to 250 °C at a rate of 10 °C/min; and finally maintaining it at 250 °C for 5 min. The mass scanning range was 35~500 *m*/*z*. The ionization (EI mode) voltage was 70 eV, the ion source temperature was 250 °C, the interface transmission temperature was 280 °C, and the fourth-stage rod temperature was 150 °C.

### 4.4. Qualitative Analysis of Volatile Flavor Components

The chromatographic and mass spectrometry data of volatile flavor components were analyzed using Agilent MassHunter Qualitative Analysis software (10.0.10305.0). The NIST 14.0 and NIST 20.0 databases were synchronously searched for qualitative analysis, and based on retention time, the compounds with a matching degree greater than 80 (the highest matching degree is 100) were identified as volatile flavor substances.

The LRI value of the flavor substance was calculated according to the formula [48]:

(1) $$\mathrm{LRI}=100\times n+\frac{100\;(t_{a}-t_{n})}{t_{n+1}-t_{n}}$$

where t_a_ is the retention time of sample a; t_n_ is the retention time of n-alkane C_n_; t_n+1_ is the retention time of C_n+1_, a normal alkane. The retention time of sample a falls between C_n_ and C_n+1_. 

### 4.5. Quantitative Determination of Volatile Flavor Components

The internal standard method was used for the quantitative determination of volatile flavor components [49]. Briefly, o-dichlorobenzene was used as the internal standard, the content of the flavor substance relative to the internal standard can be calculated according to the formula:

(2) $$W_{a}=f_{a}\times \frac{A_{a}\times m_{s}/A_{s}}{m}$$

In the formula, W_a_ is the content of volatile substance a to be tested (mg/kg); f_a_ is the quality correction factor, generally denoted as 1; A_a_ is the peak area of the volatile substance a to be tested; A_s_ is the peak area of the internal standard ortho dichlorobenzene; m_s_ is the mass of the internal standard o-dichlorobenzene; m is the mass of the substance to be tested.

### 4.6. Determination of Characteristic Volatile Flavor Components

OAV (odor activity value) was used to characterize the contribution of volatile flavor compounds [50]. In view of the fact that the analytes were semi-qualitative, here the estimated OAV (EOAV) was used, which is determined by the equation:

(3) $$EOAV=\frac{C_{i}}{T_{i}}$$

where EOAV is the estimated odor activity value of the volatile substance i, C_i_ is the content of the volatile substance i (mg/kg), and T_i_ is the olfactory threshold of the volatile substance i in water (mg/kg). The olfactory threshold of volatile flavor compounds in water was available at: https://www.vcf-online.nl/VcfCompoundSearch.cfm (accessed on 20 May 2021). The substance with an EOAV greater than or equal to 1 is determined as the main flavor component.

On the other hand, the contribution rate was used to visually characterize the contribution value of a volatile flavor component to the overall flavor. The specific calculation method for the contribution rate is the ratio of the EOAV of a single volatile flavor component to the sum of all volatile flavor components. In this study, characteristic flavor components were defined as the flavor components with a contribution rate of >3%.

### 4.7. Determination of Primary Nutrients

The content changes in the main components were studied during the 0–70 min roasting stage of passion fruit-roasted chicken. Amino acid analysis (Biochrom 30+, Biochrom Co., Ltd., Cambridge, UK) and ion chromatography (1260, Agilent, Santa Clara, CA, USA) were used to analyze the dynamic changes in flavor precursors such as free amino acids, free fatty acids, and soluble sugars in the samples during the roasting process. The infrared drying method (MA160-1CN, Sedolis Scientific Instruments GmbH, Gilching, Germany) was used for the determination of moisture content, and the Kjeldahl nitrogen method (K9840, Shandong Haineng Scientific Instrument Co., Ltd., Jinan, China) was used for the determination of protein contents.

Regarding free amino acids [51], 2.0 g of the chicken sample was added to a 50 mL volumetric flask and 20 mL of 5% sulfosalicylic acid was added, which was left to stand for 24 h. The supernatant (20 mL) was centrifuged for 15 min (6000 r/min) and was dried, adding 1 mL of sodium citrate buffer, and the amino acids were analyzed after membrane filtration (0.45 μm). The UV detection wavelengths were 570 nm and 440 nm (Pro). By comparing the retention time and peak area of various amino acid standards, the types and contents of each amino acid were determined.

Regarding nucleotides [52], 5.0 g of the passion fruit-roasted chicken meat sample was mixed with 20 mL of pre-cooled 5% perchloric acid and homogenized at 4 ° C for 2 min (7000 r/min, 20 s, 6 times). After homogenization, a freeze centrifuge was used to remove protein (4000 r/min, 4 °C, 10 min). We then collected supernatant 1 and mixed the remaining residue with 10 mL of 5% perchloric acid again. Repeating the above process, supernatant 2 was collected, and supernatants 1 and 2 were combined. After adjusting pH to 6.0 with 5 mol/L KOH solution, it was quickly placed at 4 °C and stood for 1 h to precipitate potassium perchlorate and filtered (0.22 μm) into an automatic injection bottle for testing by HPLC (1260, Agilent, USA). We then injected 5 μ L of sample and identified the nucleotide analyte from gradient elution at 254 nm. The column temperature was set to 30 °C, and mobile phase A was KH_2_PO_4_ (0.02 mol/L, pH 7). Mobile phase B used high-performance liquid chromatography-grade methanol as the eluent. For separation, we adopted the gradient elution method with a constant flow rate of 1.0 mL/min and equilibrated the sample for 30 min before elution. The elution procedure was as follows: 0–3 min, 99% A; 3~3.5 min, 99% A~95% A; 3.5~5.5 min, 95% A; 5.5~6.5 min, 95% A~85% A; 6.5~9 min, 85% A; 9–10 min, 85% A~99% A. After washing, we rinsed the needle with a mixture of ultrapure water and methanol (1:1, *v*/*v*).

For free fatty acids [53], 100 mg of passion fruit-roasted chicken was mixed well with 4 mL of the chloroform solution, centrifuged for 15 min (3500 r/min at a room temperature of 25 °C), stood for 1 h, and filtered. Adding 6 mL of the NaCl solution to the filtrate, the lower layer liquid 1 was transferred after centrifugation and the remaining liquid was mixed with 2 mL of dichloromethane. We then repeated the above operation, and the lower layer liquid 2 was aspirated. Liquids 1 and 2 were merged and dried with a nitrogen-purging instrument. GC-MS (6890A-5975C, Agilent, USA) was performed. The flow rate was 1.0 mL/min. The initial temperature was 100 °C, maintained for 5.0 min, and heated to 240 °C with a program of 4 °C/min for 15 min. The quality scanning range of MS was 30–550 *m*/*z*. 

Regarding soluble sugars [54], 50 mg of freeze-dried passion fruit-roasted chicken powder was transferred to a rotary tube, and then 700 μ 80% chromatographic grade ethanol was added, shaking at 50 °C for 2 h. Adding 700 μ of ultrapure water and centrifuging for 3 min (10,000 r/min at a room temperature of 25 °C), the supernatant was aspirated and filtered (0.22 μ) through an automatic injection vial for machine detection. The mobile phase was A: ultrapure water, B: 100 mmol/L NaOH; Injection volume of 5 μ L. The flow rate was 0.5 mL/min, and the column temperature was 30 °C. Using the gradient elution method, the specific elution procedure was as follows: 0–9 min, 95% A; 9~20 min, 95% A~0% A; 20~30 min, 0% A; 30~30.1 min, 0% A~95% A; 30.1–40 min, 95% A; 40~60 min, 95% A. 

### 4.8. Determination of Volatile Flavor Components during Storage Period

The finished roasted chicken was packaged via vacuuming and sealed in an aluminum foil bag, then stored for 30 days at 4 °C. The changes in volatile flavor components at different time points (5, 10, 15, 20, 25, and 30 days) during storage were assayed using the above method.

### 4.9. Data Processing and Analysis

Agilent MassHunter Qualitative Analysis software and NIST 14.0 and NIST 20.0 databases were used to analyze flavor substances. ANOVA analysis of variance using SPSS Statistics 26.0 and Duncan’s multiple comparison method were employed to analyze significance (*p* < 0.05). Mean and standard deviation analyses were performed using Excel 16.43, and the results are expressed as mean ± standard deviation (Mean ± SD) with n = 3.

## Data Availability

The data presented in this study are available on request from the corresponding author. The data are not publicly available due to privacy restrictions.

## Supplementary Materials

The following supporting information can be downloaded at: https://www.mdpi.com/article/10.3390/foods13142221/s1, Table S1. Identification of volatile flavor compounds in passion fruit roasted chicken; Table S2. Changes of Volatile flavor components during the processing of passion fruit roasted chicken; Table S3. Changes of main components during the processing of passion fruit roasted chicken; Table S4. Changes of free amino acids during the processing of passion fruit roasted chicken; Table S5. Changes of nucleotides during the processing of passion fruit roasted chicken; Table S6. Changes of free fatty acids during the process of passion fruit roasted chicken; Table S7. Changes of soluble saccharides during the processing of passion fruit roasted chicken; Table S8. Changes of volatile flavor components of passion fruit roasted chicken during storage; Table S9. Comparison of volatile flavor compounds between passion fruit roasted chicken and other pre-made chicken products; Table S10. Abbreviation and full name.
