# Supplementary material for: Flavoromics Analysis of Passion Fruit-Roasted Chicken
Source: Foods. 2024 Jul 15;13(14):2221. doi: 10.3390/foods13142221 (PMC11276248; doi:10.3390/foods13142221)
Supplement: Supplementary file 1 [file foods-13-02221-s001.zip › foods-3049401-supplementary.pdf]

Supplemental materials

Table S1 Identification of volatile flavor compounds in passion fruit roasted chicken

Retention time (min)	Compounds	Molecular formula	Matching degree	CAS number	LRI value	Threshold (ng/g)	Content (ng/g)	Estimated odor activity value (EOAV)	Contribution rate (%)	Odor characteristics
**Aldehydes**										
3.476	Isovaleraldehyde	$C_5H_{10}O$	83.59	590-86-3	909	2	18.66±3.31	9.33	3.15	Apple aroma, citrus flavor
4.440	valeraldehyde	$C_5H_{10}O$	90.65	110-62-3	978	8	20.80±1.51	2.60	0.88	Fruit aroma, bread aroma
6.501	hexanal	$C_6H_{12}O$	94.79	66-25-1	1028	5	57.21±6.63	11.44	3.87	Grassy and woody flavors
13.913	nonanal	$C_9H_{18}O$	98.23	124-19-6	1337	4.25	6.83±0.11	1.61	0.54	Wax fragrance, citrus fragrance, floral fragrance
15.617	3-Furfural	$C_5H_4O_2$	88.48	498-60-2	1455	8	83.65±6.53	10.46	3.53	Bread aroma, caramel flavor, roasted flavor
16.867	benzaldehyde	C_7H_6O	95.34	100-52-7	1406	300	127.57±9.79	0.43	0.14	Almond, cherry, and nut aromas
**Ketones**										
8.877	5-methyl-2-hexanone	$C_7H_{14}O$	81.22	110-12-3	1133	440	22.85±2.76	0.05	0.02	Sweet and mellow fragrance
11.545	3-hydroxy-2-butanone	$C_4H_8O_2$	93.75	513-86-0	1281	8000	58.91±7.08	0.01	0	Cream aroma, milk aroma

17.937	2-furanylacetone	$C_7H_8O_2$	87.02	6975-60-6	1562	832	14.13±1.03	0.02	0.01	Fruit flavor, spicy aroma, and small radish aroma
25.960	Ethyl Maltol	$C_7H_8O_3$	97.03	4940-11-8	1966	44	641.19±38.50	14.57	4.93	Sweet caramel flavor
**alcohols**										
9.597	2-methylbutanol	$C_5H_{12}O$	90.25	1565-80-6	1222	320	124.56±9.91	0.39	0.13	Apple white aroma and spicy taste
12.194	2-Heptanol	$C_7H_{16}O$	90.77	543-49-7	1317	100	7.63±0.71	0.08	0.03	Light oil aroma and wine aroma
12.984	Hexanol	$C_6H_{14}O$	92.73	626-93-7	1353	200	19.21±1.63	0.10	0.03	Fragrant, fruity, mellow and sweet
15.034	(S) - Linalool oxide	$C_{10}H_{18}O_2$	83.62	5989-33-3	1433	250	5.94±0.03	0.02	0.01	Floral aroma, tea aroma, and woody aroma
17.227	L-Linalool	$C_{10}H_{18}O$	98.63	126-91-0	1542	6	16.24±2.00	2.71	0.91	Lilac aroma, woody notes
20.247	Terpineol	$C_{10}H_{18}O$	92.65	8000-41-7	1667	300	7.62±1.29	0.03	0.01	Ding fragrance, pine flavor
24.279	Phenylethanol	$C_8H_{10}O$	92.12	60-12-8	1885	0.75	32.86±3.05	43.81	14.81	Gardenia flavor, lilac, rose fragrance
**Acids and esters**										
15.375	acetic acid	$C_2H_4O_2$	83.25	64-19-7	1429	6400	16.12±2.02	0	0	sour
3.101	ethyl acetate	$C_4H_8O_2$	97.55	141-78-6	883	100	50.35±9.01	0.50	0.17	Banana and apple aromas, fragrant taste

5.545	Ethyl Butyrate	$C_6H_{12}O_2$	94.45	105-54-4	1017	1	149.22±30.95	149.22	50.44	Pineapple aroma
10.090	ethyl caproate	$C_8H_{16}O_2$	94.79	123-66-0	1226	8	37.82±6.75	4.73	1.60	Pineapple aroma, fruity aroma
12.841	ethyl lactate	$C_5H_{10}O_3$	90.65	97-64-3	1327	50000	137.36±19.04	0	0	Fatty taste, bitter taste
13.993	1-Methylbutyrate hexyl ester	$C_{11}H_{22}O_2$	87.03	10632-13-0	1388	1110	4.9±0.22	0	0	Fruit aroma, light aroma, wax aroma
14.376	Hexyl butyrate	$C_{10}H_{20}O_2$	92.36	2639-63-6	1407	203	6.57±0.31	0.03	0.01	Fruit aroma, apricot aroma, ester aroma, vegetable aroma, and pineapple aroma
14.801	Ethyl octanoate	$C_{10}H_{20}O_2$	91.19	106-32-1	1439	0.69	4.67±0.27	6.77	2.29	Pineapple aroma, apple aroma, brandy aroma
16.691	3-Hydroxy butyrate ethyl ester	$C_6H_{12}O_3$	81.56	5405-41-4	1508	2500	14.65±0.67	0.01	0	Fruit aroma, grape aroma, light aroma
**Hydrocarbons**										
6.771	3-carene	$C_{10}H_{16}$	90.39	13466-78-9	1070	44	16.12±2.02	0.37	0.12	Turpentine fragrance
7.687	γ-terpinene	$C_{10}H_{16}$	93.56	99-85-4	1244	1000	46.17±3.00	0.05	0.02	Sweet pine oil, fennel sweet
8.127	α-phellandrene	$C_{10}H_{16}$	88.58	99-83-2	1161	40	4.65±0.33	0.12	0.04	Black pepper flavor, mint flavor
8.193	β-myrcene	$C_{10}H_{16}$	82.61	123-35-3	1166	42	6.44±0.67	0.15	0.05	Light and creamy aroma, tropical fruit

										flavor
9.001	(+)-Limonene	$C_{10}H_{16}$	89.89	5989-27-5	1187	210	31.50±6.09	0.15	0.05	Fragrant, floral, and citrus flavors
10.463	ocimene	$C_{10}H_{16}$	90.6	13877-91-3	1203	34	4.36±0.72	0.13	0.04	Flower fragrance, grass fragrance, orange fragrance
10.735	styrene	C_8H_8	95.62	100-42-5	1249	730	7.70±0.75	0.01	0	Resin flavor, floral aroma
18.095	Guaiacol	$C_{15}H_{24}$	88.02	90-05-1	1599	0.22	5.71±0.62	25.95	8.77	Smoky, vanilla, and woody flavors
18.241	β-caryophyllene	$C_{15}H_{24}$	97.06	87-44-5	1600	64	151.78±28.36	2.37	0.80	Dingxiang and turpentine flavors
19.704	Z. Z, Z-1,5,9,9-tetramethyl-1,4,7-cycloundecanetriene	$C_{15}H_{24}$	88.32	6753-98-6	1578	40	6.53±0.39	0.16	0.06	Hop flavor, clove aroma
5.621	toluene	C_7H_8	83.63	108-88-3	1033	140	50.63±3.47	0.36	0.12	Aromatic odor
7.370	ethylbenzene	C_8H_{10}	86.35	100-41-4	1123	16	8.98±0.74	0.56	0.19	Aromatic odor
7.571	P-Xylene	C_8H_{10}	86.15	106-42-3	1137	530	6.03±0.84	0.01	0	Aromatic flavor
10.604	1-Pentene	$C_{10}H_5$	83.41		1227	430	4.30±0.53	0.01	0	tartness

10.941	Anisin	$C_{10}H_{14}$	84.32	109-67-1643-28-7	1803	4	5.57±0.67	1.39	0.47	Anise aroma, spice flavor
**Ethers**										
3.673	dimethyl ether	C_2H_6O	86.43	115-10-6	283	560	797.03±31.85	1.42	0.48	Aromatic odor
22.764	Anethole	$C_{10}H_{12}O$	81.35	104-46-1	1802	1.8	4.95±0.39	2.75	0.93	Sweet fennel aroma, spices, licorice flavor
**Furan**										
9.997	2-Pentylfuran	$C_9H_{14}O$	87.76	3777-69-3	1247	6	5.67±0.27	0.95	0.32	Vegetable flavor and meat aroma

Table S2 Changes of Volatile flavor components during the processing of passion fruit roasted chicken

Content（ng/g）		Different processing stages						
Retention time (min)	Compounds	Raw chicken meat	Cured meat	Roasting for 15 minutes	Roasting for 30 minutes	Roasting for 45 minutes	Roasting for 60 minutes	Finished product
aldehydes								
3.476	Isovaleraldehyde	0[d]	0[d]	6.52±0.99[c]	13.02 ±2.59[b]	17.18±3.07[ab]	17.91±3.98[a]	18.66±3.31[a]
4.440	valeraldehyde	11.23±0.68[e]	1.27±0.56[f]	10.19±0.64[e]	35.58±1.13[a]	29.88±0.98[b]	24.45±0.80[c]	20.80±1.51[d]
6.501	hexanal	240.86±11.46[ab]	23.19±3.69[f]	114.43±10.18[d]	244.14±23.75[a]	221.38±12.03[b]	169.16±8.56[c]	57.21±6.63[e]
6.960	3-Hydroxybutyraldehyde	0[c]	0[b]	0[b]	13.17±2.56[a]	0[b]	0[b]	0[b]
8.115	3-Methylglutaraldehyde	1.25±0.04[a]	0[b]	0[b]	0[b]	0[b]	0[b]	0[b]
8.980	Heptaldehyde	0[c]	0[c]	0[c]	20.56±2.06[a]	11.61±2.55[b]	0[c]	0[c]
9.811	2-hexenal	1.67±0.32[a]	0[b]	0[b]	0[b]	0[b]	0[b]	0[b]
11.492	octanal	6.83±0.69[b]	1.96±0.14[d]	3.60±0.25[c]	12.70±1.32[a]	0[e]	0[e]	0[e]
13.913	nonanal	6.35±0.42[c]	8.41±0.36[b]	6.44±1.00[c]	25.30±2.00[a]	8.72±0.21[b]	8.44±0.11[b]	6.83±0.11[c]

14.748	Trans-2-octenal	5.71±0.87^{a}	0^{b}	0^{b}	0^{b}	0^{b}	0^{b}	0^{b}
15.412	3-methylthiopropanal	0^{b}	0^{b}	0^{b}	0^{b}	0^{b}	4.47±0.57^{a}	0^{b}
15.617	3-Furfural	0^{e}	0^{e}	48.69±4.42^{d}	107.29±6.99^{a}	67.65±5.52^{c}	59.24±3.91^{c}	83.65±6.53^{b}
16.867	benzaldehyde	0^{f}	7.41±0.38^{e}	62.81 ±10.54^{d}	140.29±7.28^{a}	115.53±12.51^{bc}	101.34±6.07^{c}	127.57±9.79^{ab}
19.341	phenylacetaldehyde	0^{e}	0^{e}	4.74±0.13^{d}	14.35±0.68^{a}	7.08±0.11^{b}	5.73±0.73^{c}	0^{e}
21.936	3-isopropylbenzaldehyde	0^{b}	0^{b}	0^{b}	6.26±1.02^{a}	0^{b}	0^{b}	0^{b}
28.984	Piperonal	0^{e}	0.97±0.23^{d}	1.85±0.18^{c}	6.16±0.07^{a}	4.13±0.14^{b}	0^{e}	0^{e}
	Subtotal	273.90±59.76^{d}	43.21±6.07^{e}	259.27±32.08^{d}	638.82±67.53^{a}	483.16±59.89^{b}	390.74±47.54^{c}	314.72±37.58^{cd}
Ketones								
3.258	2-butanone	0^{c}	0^{c}	0^{c}	10.93±0.39^{b}	13.19±1.45^{a}	14.31±0.78^{a}	0^{c}
8.877	5-methyl-2-hexanone	0^{b}	0^{b}	0^{b}	0^{b}	0^{b}	0^{b}	22.85±2.76^{a}
11.545	3-hydroxy-2-but	0^{e}	0^{e}	4.55±0.55^{d}	13.99±0.99^{d}	88.79±11.61^{a}	45.31±5.01^{c}	58.91±7.08^{b}

	anone							
12.672	Methyl heptenone	0[b]	0[b]	1.78±0.23[a]	0[b]	0[b]	0[b]	0[b]
17.944	2-furanylacetone	0[c]	0[c]	0[c]	0[c]	0[c]	9.06±0.72[b]	14.13±1.03[a]
25.443	4-Camphenyl-2-butanone	0[b]	0[b]	0[b]	0[b]	4.36±0.92[a]	0[b]	0[b]
25.960	Ethyl Maltol	0[f]	143.21±16.05[e]	264.20±22.00[d]	1147.12±54.50[a]	724.59±45.55[b]	446.46±35.77[c]	641.19±38.50[b]
	Subtotal	0[d]	143.21±54.13[c]	270.53±99.47[c]	1172.04±432.04[a]	830.93±269.09[ab]	515.14±165.21[bc]	737.08±237.24[ab]
alcohols								
9.597	2-methylbutanol	0[e]	34.48±1.82[d]	58.43±4.97[c]	135.53±14.55[a]	95.24±15.09[b]	91.16±7.52[b]	124.56±9.91[a]
10.578	amyl alcohol	33.09±4.28[a]	0[c]	0[c]	9.57±0.92[b]	0[c]	0[c]	0[c]
12.194	2-Heptanol	0[d]	2.35±0.39[c]	5.01±0.84[b]	8.53±0.55[a]	6.09±0.55[b]	5.50±0.50[b]	7.63±0.71[a]
12.257	3-Penten-2-ol	0[b]	0[b]	1.44±0.47[a]	0[b]	0[b]	0[b]	0[b]
12.984	hexyl alcohol	30.09±5.81[b]	47.51±7.51[a]	17.10±1.53[c]	30.62±6.50[b]	32.82±2.50[b]	20.59±1.54[c]	19.21±1.63[c]
13.721	3-Hexene-1-ol	0[c]	1.44±0.44[b]	2.85±0.54[a]	0[c]	0[c]	3.57±0.35[a]	0[c]
14.997	α-Methyl- α -	0[b]	1.98±0.08[a]	0[b]	0[b]	0[b]	0[b]	0[b]

	4-methyl-3-pentenyl glycidyl							
15.034	Linalool oxide	0[e]	0[e]	4.52±0.25[d]	7.17±0.17[a]	6.58±0.19[b]	5.90±0.10[c]	5.94±0.03[c]
15.161	3-octanol	18.47±1.51[a]	2.37±0.38[c]	2.30±0.28[c]	5.82±0.19[b]	6.12±0.31[b]	3.17±0.20[c]	0[d]
15.259	Heptanol	2.59±0.09[a]	1.08±0.03[a]	0[b]	0[b]	0[b]	0[b]	0[b]
15.616	borneol	0[b]	3.84±0.88[a]	0[b]	0[b]	0[b]	0[b]	0[b]
17.227	L-Linalool	0[e]	6.42±0.22[d]	14.37±0.48[c]	29.44±1.34[a]	20.42±1.98[b]	16.14±2.09[c]	16.24±2.00[c]
19.613	3-furanmethanol	0[c]	5.33±0.31[a]	3.34±0.55[b]	0[c]	0[c]	3.11±0.12[b]	0[c]
20.247	Terpineol	0[e]	3.21±0.15[d]	7.32±0.14[c]	13.87±1.47[a]	10.16±1.86[b]	7.39±1.12[c]	7.62±1.29[c]
23.654	Phenylpropanediol	0[d]	2.61±0.15[c]	3.66±0.35[b]	7.29±0.33[a]	0[d]	2.60±0.21[c]	0[d]
24.279	Phenylethanol	0[f]	10.29±1.00[e]	15.23±0.93[de]	41.38±6.01[a]	24.78±2.48[c]	18.88±3.00[d]	32.86±3.05[b]
	Subtotal	84.24±11.27[d]	122.91±13.48[cd]	135.57±14.46[c]	289.22±33.82[a]	202.21±24.28[b]	178.01±22.40[b]	214.06±31.11[b]
Acids and esters								

3.101	ethyl acetate	0^{d}	0^{d}	19.76±2.55^{c}	45.41±4.01^{a}	29.24±4.34^{bc}	30.31±5.05^{b}	50.35±9.01^{a}
3.258	1,2,4-benzenetricarboxylic acid-1,2-methyl ester	0^{c}	0.83±0.02^{b}	4.03±0.07^{a}	0^{c}	0^{c}	0^{c}	0^{c}
4.553	Methyl Butyrate	0^{d}	3.37±0.36^{c}	10.74±1.77^{a}	11.88±2.13^{a}	6.26±0.43^{b}	7.27±0.07^{b}	0^{d}
5.545	Ethyl Butyrate	0^{d}	38.41±3.95^{c}	113.63±7.50^{b}	219.22±16.96^{a}	136.12±23.51^{b}	141.83±22.01^{b}	149.22±30.95^{b}
8.983	methyl hexanoate	10.46±0.61^{a}	2.41±0.28^{c}	9.82±0.81^{ab}	0^{d}	8.63±0.62^{b}	11.1±0.95^{a}	0^{d}
10.090	ethyl caproate	0^{d}	12.63±2.52^{c}	33.11±4.55^{b}	58.41±7.96^{a}	41.85±7.03^{b}	34.62±2.55^{b}	37.82±6.75^{b}
12.372	Vinyl hexanoate ester	19.36±3.00^{a}	0^{c}	2.19±0.40^{b}	0^{c}	4.08±0.71^{b}	0^{c}	0^{c}
12.841	ethyl lactate	0^{e}	29.63±5.42^{d}	53.71±5.49^{c}	129.08±11.14^{a}	86.69±5.55^{b}	72.27±6.95^{b}	137.36±19.04^{a}
13.850	Methyl octanoate	0^{e}	1.73±0.09^{d}	4.83±0.50^{b}	6.81±0.15^{a}	0^{e}	2.75±0.23^{c}	0^{e}
13.993	1-Methylbutyrate hexyl	0^{f}	1.93±0.15^{e}	2.74±0.20^{e}	8.96±0.23^{c}	5.95±0.37^{d}	23.81±0.11^{b}	34.90±0.22^{a}

	ester							
14.376	Hexyl butyrate	0^{d}	$2.54±0.23^{c}$	$3.09±0.42^{bc}$	$7.44±1.31^{a}$	$6.68±0.92^{b}$	$4.19±0.24^{b}$	$6.57±0.31^{b}$
14.801	Ethyl octanoate	0^{e}	$3.62±0.59^{d}$	$7.28±0.81^{c}$	$11.70±1.45^{a}$	$8.83±0.86^{b}$	$6.49±0.50^{c}$	$4.67±0.27^{d}$
15.375	acetic acid	$1.29±0.15^{b}$	0^{c}	0^{c}	$14.97±2.22^{a}$	0^{c}	0^{c}	$16.12±2.02^{a}$
16.691	ethyl 3-hydroxybutyrate	0^{f}	$2.50±0.28^{e}$	$4.87±0.13^{d}$	$12.09±0.80^{b}$	$7.27±0.59^{c}$	$7.76±0.11^{c}$	$14.65±0.67^{a}$
17.449	octyl formate	$2.04±0.65^{a}$	$1.28±0.22^{a}$	0^{b}	0^{b}	0^{b}	0^{b}	0^{b}
18.020	1-Methylhexyl hexanoate	0^{d}	$1.38±0.10^{c}$	$2.24±0.23^{b}$	0^{d}	$3.67±0.46^{a}$	0^{d}	0^{d}
18.516	Hexyl Hexanoate	0^{d}	$2.36±0.21^{c}$	$4.03±0.56^{b}$	0^{d}	$8.30±0.33^{a}$	$3.70±0.17^{b}$	0^{d}
19.077	ethyl decanoate	0^{e}	$2.17±0.61^{d}$	$4.11±0.89^{bc}$	$8.81±0.82^{a}$	$5.11±0.44^{b}$	$3.48±0.29^{c}$	0^{e}
20.146	2-Methylpropanoate ethyl ester	0^{c}	0^{c}	0^{c}	$6.14±1.12^{a}$	0^{c}	$2.37±0.79^{b}$	0^{c}
23.04	hexanoi	0^{c}	$2.69±0.88^{b}$	0^{c}	$13.60±3.01^{a}$	0^{c}	0^{c}	0^{c}

1	c acid							
	Subtotal	33.15±4.79^{f}	109.48±10.22^{e}	280.18±26.90^{d}	554.52±54.26^{a}	358.68±34.54^{bc}	331.95±34.26^{cd}	421.66±43.99^{b}
Hydrocarbons								
5.621	toluene	10.59±2.26^{cd}	4.86±0.25^{d}	14.81±0.91^{c}	47.37±2.94^{ab}	40.72±7.50^{b}	40.36±7.00^{b}	50.63±3.47^{a}
6.771	3-carene	0^{c}	6.68±1.09^{b}	6.77±0.35^{b}	50.95±5.03^{a}	49.75±8.49^{a}	9.63±1.40^{b}	8.66±1.30^{b}
6.844	β-pinene	0^{e}	0^{e}	17.79±2.46^{b}	6.53±1.27^{d}	11.96±2.02^{c}	25.69±4.50^{a}	0^{e}
7.370	ethylbenzene	5.39±0.51^{c}	0^{d}	0^{d}	11.92±2.24^{a}	0^{d}	4.98±0.84^{c}	8.98±0.74^{b}
7.571	para-xylene	8.46±0.94^{b}	0^{f}	1.62±0.18^{e}	12.21±1.16^{a}	7.10±0.80^{bc}	5.43±0.48^{d}	6.03±0.84^{cd}
7.611	O-Xylene	5.08±1.01^{c}	0^{d}	0^{d}	10.65±1.78^{b}	8.21±0.80^{b}	14.39±1.56^{a}	0^{d}
7.687	γ-Terpinene	0^{c}	0^{c}	25.59±3.56^{b}	0^{c}	0^{c}	40.22±7.00^{a}	46.17±3.00^{a}
7.772	m-xylene	10.11±1.01^{a}	0^{b}	0^{b}	0^{b}	0^{b}	0^{b}	0^{b}
8.127	α-phellandrene	0^{d}	0^{d}	1.60±0.28^{c}	0^{d}	0^{d}	0^{d}	4.65±0.33^{a}
8.193	β-myrcene	0^{b}	0^{b}	0^{b}	0^{b}	0^{b}	0^{b}	6.44±0.67^{a}
9.001	（+）-Limonene	6.81±0.82^{c}	5.92±0.65^{c}	20.31±4.00^{b}	26.68±5.56^{ab}	32.82±5.91^{a}	28.77±8.06^{ab}	31.50±6.09^{a}
10.387	α-pinene	0^{b}	0^{b}	1.71±0.08^{a}	0^{b}	0^{b}	0^{b}	0^{b}
10.463	ocimene	0^{b}	0^{b}	0^{b}	0^{b}	0^{b}	0^{b}	4.36±0.72^{a}

10.550	1-chloropentane	0^{d}	$6.42±0.44^{c}$	$6.30±0.40^{c}$	0^{d}	$13.61±1.23^{a}$	$8.53±0.91^{b}$	0^{d}
10.604	pentene	0^{b}	0^{b}	0^{b}	0^{b}	0^{b}	0^{b}	$4.30±0.53^{a}$
10.735	styrene	$8.69±0.71^{b}$	$0.93±0.05^{e}$	$2.80±0.24^{d}$	$13.57±1.49^{a}$	$8.45±0.67^{b}$	$4.72±0.30^{c}$	$7.70±0.75^{b}$
10.941	Orthocymbidium hydrocarbon	0^{c}	0^{c}	$3.48±0.29^{b}$	0^{c}	0^{c}	$4.72±0.31^{a}$	$5.57±0.67^{a}$
10.978	Umbrella hydrocarbon	$1.53±0.05^{a}$	0^{b}	0^{b}	0^{b}	0^{b}	0^{b}	0^{b}
11.041	4-isopropyltoluene	0^{c}	$1.04±0.03^{b}$	0^{c}	0^{c}	$5.55±0.72^{a}$	0^{c}	0^{c}
11.268	1,2,4-trimethylbenzene	$1.34±0.21^{a}$	0^{b}	0^{b}	0^{b}	0^{b}	0^{b}	0^{b}
11.303	1,2,3,4,5-pentamethylcyclopentadiene	0^{d}	0^{d}	$1.51±0.27^{c}$	0^{d}	$3.70±0.42^{a}$	$2.68±0.48^{b}$	0^{d}
18.095	Guaiacol	0^{e}	$3.68±0.44^{d}$	$5.19±0.34^{c}$	$11.61±1.53^{a}$	$8.60±0.86^{b}$	$4.46±0.31^{cd}$	$5.71±0.62^{c}$
18.24	β-caryop	0^{e}	$126.29±17.94^{d}$	$186.46±36.99^{c}$	$409.07±29.70^{a}$	$302.11±33.47^{b}$	$137.97±21.51^{cd}$	$151.78±28.36^{cd}$

19.73<br>4	hyllene Z. Z, Z-1,5,9, 9-tetramethyl-1,4,7-cycloundecane triene	0[e]	6.34±0.52[d]	10.45±0.67[c]	17.66±1.51[a]	14.41±1.83[b]	6.75±0.48[d]	6.53±0.39[d]
20.68<br>7	Decahydro-4a-methyl-1-methylene-7-(1-methylethylidene) -(4ar trans) naphthalene	0[e]	6.22±0.44[d]	9.17±0.40[c]	18.73±1.39[a]	15.64±0.68[b]	6.77±0.46[d]	0[e]
20.78<br>5	Decahydro-4a-methyl-1-methylene-7-(1-methylvinyl) - [4ar -(4a α ,	0[d]	5.02±0.62[c]	6.37±0.39[c]	17.82±1.61[a]	13.17±1.21[b]	5.46±0.49[c]	0[d]

	seven α, 8a β）] naphthalene							
25.446	Caryophyllene oxide	0^{c}	1.59±0.38^{b}	3.29±0.88^{a}	0^{c}	0^{c}	0^{c}	0^{c}
	Subtotal	58.00±3.63^{e}	174.99±24.08^{d}	325.22±35.54^{c}	654.77±78.14^{a}	535.80±57.88^{b}	351.53±27.60^{c}	349.01±30.80^{c}
Ethers								
3.673	dimethyl ether	0^{f}	142.13±35.87^{e}	224.91±37.04^{d}	689.17±30.00^{b}	388.31±65.35^{c}	346.19±43.49^{c}	797.03±31.85^{a}
22.764	Anethole	0^{f}	4.04±0.25^{e}	7.00±0.23^{c}	14.34±1.49^{a}	10.67±1.05^{b}	5.72±0.25^{cd}	4.95±0.39^{de}
	Subtotal	0^{e}	146.17±97.64^{d}	231.91±154.09^{c}	703.51±477.18^{a}	398.98±267.03^{b}	351.91±240.75^{b}	801.98±560.09^{a}
Furan								
9.997	2-Pentyl furan	3.03±0.36^{c}	1.49±0.17^{d}	0^{e}	7.29±0.50^{a}	6.84±0.63^{a}	0^{e}	5.67±0.27^{b}
	Total	452.32±25.95^{f}	741.46±25.60^{e}	1502.68±44.39^{d}	4020.17±149.70^{a}	2816.60±95.44^{b}	2119.28±65.39^{c}	2844.18±110.75^{b}

Note: Data with different letters (a, b, c, d, e) in the same row represent significant differences between different processing stages ($p<0.05$).

Table S3 Changes of main components during the processing of passion fruit roasted chicken

Contents（mg/100g）	Different processing stages						
Main compone	Raw chicken	After curing	Baking for 15	Baking for 30	Baking for 45	Baking for 60	Finished products

nts			min	min	min	min	
Water	73.42±0.62^{a}	74.41±1.07^{a}	67.99±0.41^{b}	66.03±0.14^{c}	63.14±0.29^{d}	59.89±0.22^{e}	53.55±0.44^{f}
Proteins	70.77±0.46^{a}	52.32±0.48^{e}	54.21±0.64^{cd}	56.48±0.69^{b}	54.76±0.96^{c}	53.35±0.21^{de}	53.36±0.43^{de}
Fats	19.36±0.30^{a}	15.45±0.22^{c}	11.85±0.81^{e}	14.07±0.63^{d}	17.82±0.12^{b}	17.12±0.38^{b}	12.23±0.63^{e}

Note: Data with different letters (a, b, c, d, e) on the same line represent significant differences in the content of main components at different stages ($p<0.05$).

Table S4 Changes of free amino acids during the processing of passion fruit roasted chicken.

Content (mg/100g)	Different processing stages						
Name of amino acids	Raw chicken	After curing	Baking for 15 min	Baking for 30 min	Baking for 45 min	Baking for 60 min	Finished product
Aspartate	32.13±0.85^{e}	36.17±0.67^{bc}	35.80±1.20^{bc}	38.30±0.90^{a}	33.30±0.36^{de}	34.73±0.80^{cd}	36.61±1.59^{ab}
Threonine	20.26±2.06^{ab}	17.45±1.60^{b}	19.80±0.98^{b}	24.17±3.45^{a}	19.26±3.56^{b}	18.13±0.88^{b}	17.42±1.50^{b}
Serine	34.12±0.95^{d}	46.34±1.37^{a}	43.11±1.06^{ab}	42.71±1.27^{b}	23.28±1.73^{e}	37.39±2.22^{c}	43.49±2.93^{ab}
Glutamate	63.46±3.38^{a}	60.34±2.87^{ab}	58.26±3.99^{ab}	63.70±4.32^{a}	58.93±1.72^{ab}	56.50±2.74^{b}	55.64±1.56^{b}
Glycine	25.17±0.82^{a}	20.84±0.88^{bc}	18.39±0.95^{d}	22.41±1.46^{b}	21.55±1.71^{b}	19.28±0.93^{cd}	18.29±0.50^{d}
Alanine	35.79±0.58^{a}	32.64±3.38^{abc}	28.48±2.49^{cd}	35.33±2.20^{ab}	31.43±1.51^{bc}	26.28±2.01^{d}	29.82±2.51^{cd}
Cystine	1.29±0.04^{bc}	1.20±0.12^{c}	1.20±0.05^{c}	1.30±0.06^{bc}	2.30±0.11^{a}	1.39±0.05^{b}	1.03±0.09^{d}
Valine	40.95±1.76^{b}	38.90±2.39^{b}	39.37±1.48^{b}	45.59±2.26^{a}	39.33±2.11^{b}	38.64±2.10^{b}	30.75±1.28^{c}
Methionine	20.41±1.02^{ab}	20.91±0.97^{ab}	21.81±1.50^{ab}	23.14±0.90^{a}	21.18±2.86^{ab}	19.40±3.47^{b}	9.70±0.37^{c}

Isoleucine	18.67±1.11^{b}	16.09±0.86^{c}	17.59±1.30^{bc}	21.82±1.13^{a}	17.58±1.10^{bc}	15.64±1.32^{c}	12.08±0.86^{d}
Leucine	34.81±3.34^{b}	35.62±2.09^{b}	36.67±1.00^{b}	40.86±1.83^{a}	34.66±1.93^{b}	34.50±0.77^{b}	28.79±2.59^{c}
Tyrosine	23.45±2.24^{ab}	23.30±2.37^{ab}	22.31±1.04^{b}	26.90±1.35^{a}	21.41±3.83^{b}	19.92±2.89^{b}	21.07±1.73^{b}
Phenylalanine	22.03±1.98^{ab}	24.33±1.08^{a}	22.49±1.51^{ab}	24.67±1.54^{a}	19.40±1.38^{b}	21.24±2.07^{ab}	21.06±2.96^{ab}
Histidine	58.41±2.96^{a}	30.58±2.57^{b}	22.91±1.09^{c}	58.63±2.69^{a}	29.25±1.31^{b}	29.43±1.27^{b}	25.32±2.11^{c}
Lysine	37.17±1.39^{a}	27.35±1.65^{d}	31.03±0.85^{bc}	39.29±2.83^{a}	33.20±1.04^{b}	30.26±1.00^{c}	24.73±1.16^{d}
Arginine	57.74±3.50^{ab}	46.33±2.00^{cd}	64.09±4.74^{a}	62.28±4.15^{a}	52.09±3.35^{bc}	48.02±2.20^{cd}	44.59±3.60^{d}
Proline	19.39±0.89^{b}	26.82±1.72^{a}	24.61±1.45^{a}	26.14±1.86^{a}	21.43±2.44^{b}	20.58±1.66^{b}	24.98±1.98^{a}
Total flavor amino acids content	545.25±16.06^{b}	505.19±13.61^{c}	507.94±15.15^{c}	597.25±16.25^{a}	479.56±13.30^{d}	471.33±13.02^{d}	445.28±13.41^{de}

Note: Data with different letters (a, b, c, d, e) on the same line represent significant differences in the content of free amino acids at different stages ($p<0.05$).

Table S5 Changes of nucleotides during the processing of passion fruit roasted chicken

Content (mg/100g)	Different processing stages						
Name of nucleotides	Raw chicken	After curing	Baking for 15 min	Baking for 30 min	Baking for 45 min	Baking for 60 min	Finished product
5'-ATP	39.58±1.21^{a}	40.17±1.06^{a}	15.93±0.17^{b}	7.05±0.49^{c}	8.04±0.11^{c}	0^{f}	0^{f}
5'-ADP	6.59±0.22^{e}	3.93±0.28^{f}	14.25±0.77^{c}	15.66±0.67^{b}	12.53±0.65^{d}	12.97±0.78^{cd}	6.59±0.22^{e}
5'-AMP	2.40±0.09^{c}	0.71±0.03^{d}	8.15±0.12^{ab}	8.34±0.21^{a}	8.25±0.26^{a}	8.69±0.29^{a}	7.98±0.31^{b}
5'-IMP	10.50±0.50^{d}	8.86±0.07^{e}	56.92±2.76^{c}	62.99±1.58^{b}	73.09±4.11^{a}	77.09±2.12^{a}	62.70±1.10^{b}
5'-GMP	8.54±0.32^{a}	2.14±0.09^{d}	1.21±0.06^{e}	6.34±0.42^{c}	7.87±0.85^{b}	7.97±0.33^{ab}	6.07±0.35^{c}

I	41.84±2.61^{ab}	28.30±2.20^{d}	39.70±1.56^{bc}	35.19±4.03^{c}	42.45±1.82^{ab}	46.51±3.31^{a}	45.12±2.90^{a}
Hx	145.49±5.52^{bc}	98.39±5.19^{d}	129.86±8.39^{c}	131.78±7.83^{c}	156.96±12.97^{ab}	169.17±12.00^{a}	157.17±14.00^{ab}
Flavor nucleotides	21.44±4.23^{c}	11.71±4.35^{d}	66.28±30.36^{b}	77.67±32.15^{ab}	89.21±37.55^{a}	93.75±39.70^{a}	76.75±32.16^{ab}

Note: Data with different letters (a, b, c, d, e) on the same line represent significant differences in nucleotide content at different processing stages ($p<0.05$).

Table S6 Changes of free fatty acids during the process of passion fruit roasted chicken

Contents (g/100g)	Different processing stages						
Names of fatty acids	Raw chicken	After curing	Baking for 15 min	Baking for 30 min	Baking for 45 min	Baking for 60 min	Finished product
Capric acid（C10:0）	0^{e}	0.44±0.02^{c}	0.31±0.03^{d}	0.08±0.02^{e}	0.06±0.02^{e}	0.89±0.03^{b}	1.48±0.03^{a}
Undecanoic acid（C11:0）	0^{b}	0^{b}	0.06±0.02^{a}	0^{b}	0^{b}	0^{b}	0^{b}
Lauric acid（C12:0）	1.20±0.29^{e}	6.52±0.18^{c}	5.66±0.29^{d}	5.30±0.17^{d}	5.06±0.12^{d}	7.52±0.10^{b}	12.59±0.70^{a}
Tridecanoic acid（C13:0）	0.26±0.03^{d}	0.86±0.03^{b}	1.14±0.08^{a}	0.92±0.03^{b}	1.22±0.05^{a}	0.98±0.05^{b}	0.51±0.02^{c}
Myristate（C14:0）	27.97±0.13^{d}	29.46±0.59^{cd}	25.60±0.74^{e}	29.71±0.67^{cd}	30.67±0.86^{c}	39.35±0.67^{b}	48.37±0.69^{a}
Myristoleic acid（C14:1）	5.97±0.09^{c}	6.34±0.04^{c}	6.75±0.13^{b}	6.04±0.08^{c}	7.91±0.22^{a}	8.20±0.19^{a}	7.02±0.12^{b}
Pentadecanoic acid（C15:0）	6.91±0.12^{ab}	5.35±0.57^{d}	4.96±0.17^{d}	6.34±0.11^{c}	7.26±0.60^{a}	7.95±0.39^{a}	7.28±0.47^{a}
Pentadecenoic acid（C15:1）	1.55±0.09^{e}	1.71±0.08^{cd}	1.95±0.06^{ab}	1.87±0.08^{ab}	2.05±0.07^{a}	1.82±0.03^{bc}	1.67±0.02^{d}
Palmitic acid（C16:0）	641.56±23.57^{a}	323.40±35.48^{e}	374.37±25.45^{d}	361.11±9.34^{de}	414.35±26.55^{c}	501.57±10.34^{b}	446.59±10.77^{c}
Palmitoleic acid（C16:1）	192.43±7.54^{a}	119.59±6.80^{d}	149.05±7.86^{c}	122.27±3.84^{d}	175.46±6.15^{b}	194.27±5.59^{a}	118.25±7.62^{d}
Margaric acid（C17:0）	10.52±0.52^{a}	6.07±0.16^{d}	5.67±0.24^{d}	7.55±0.12^{c}	7.71±0.17^{c}	8.86±0.28^{b}	7.61±0.36^{c}
Heptadecenoic acid（C17:1）	15.53±0.54^{a}	8.07±0.09^{e}	7.81±0.17^{e}	10.53±0.48^{bc}	10.43±0.39^{c}	11.50±0.56^{b}	9.15±0.08^{d}
Stearic acid（C18:0）	466.58±15.14^{a}	225.30±5.22^{d}	228.00±7.82^{d}	283.94±5.23^{c}	278.38±9.14^{c}	362.26±9.21^{b}	300.46±9.73^{c}
Trans 9-octadecenoic acid（C18:1T）	12.45±0.85^{a}	7.50±0.19^{c}	9.66±0.13^{b}	8.07±0.16^{c}	9.60±0.33^{b}	10.54±0.63^{b}	12.68±0.71^{a}
Oleic acid（C18:1）	1760.00±49.47^{a}	878.89±31.25^{f}	1065.65±24.99^{de}	980.96±19.63^{e}	1184.37±16.78^{c}	1442.20±45.21^{b}	1114.08±25.90^{cd}

Trans linoleic acid（C18:2T）	2.24±0.12^{cd}	2.47±0.12^{bc}	2.75±0.27^{ab}	2.54±0.17^{bc}	3.01±0.18^{a}	2.64±0.14^{b}	1.99±0.13^{d}
Linoleic acid（C18:2）	2061.36±29.39^{a}	1004.91±25.09^{e}	1133.73±34.03^{d}	1145.08±20.54^{d}	1192.25±11.53^{d}	1525.61±16.83^{b}	1366.80±20.34^{c}
Arachidic acid（C20:0）	7.77±0.16^{a}	3.83±0.14^{d}	5.58±0.25^{bc}	4.15±0.12^{d}	5.22±0.10^{c}	5.80±0.11^{b}	5.46±0.14^{bc}
r-Linolenic acid（C18:3）	12.36±0.16^{bc}	9.97±0.27^{d}	11.76±0.48^{c}	12.71±0.29^{b}	13.12±0.13^{b}	17.25±0.16^{a}	11.62±0.40^{c}
11- Eicosenoic acid（C20:1）	19.46±1.16^{a}	9.76±1.14^{c}	10.92±0.32^{c}	10.21±1.09^{c}	13.50±2.17^{b}	14.69±0.18^{b}	12.35±1.12^{b}
Linolenic acid（C18:3）	204.45±13.05^{a}	93.45±5.01^{d}	106.05±6.67^{cd}	110.63±3.68^{cd}	119.08±4.64^{c}	143.72±2.64^{b}	119.21±4.10^{c}
Heneicosanoic acid（C21:0）	0.84±0.20^{d}	1.42±0.09^{c}	1.91±0.33^{a}	1.50±0.03^{c}	1.94±0.07^{a}	1.64±0.02^{ab}	0.72±0.03^{d}
11,14-icosidiene酸（C20:2）	26.14±2.99^{a}	12.79±1.03^{c}	13.03±0.07^{c}	17.40±2.40^{bc}	15.44±1.65^{bc}	18.67±0.70^{b}	16.90±1.13^{bc}
Behenic acid（C22:0）	4.07±0.45^{a}	2.40±0.05^{d}	3.46±0.34^{ab}	2.67±0.07^{cd}	3.28±0.23^{bc}	3.52±0.07^{ab}	2.93±0.09^{bc}
Cis 8,11,14-eicosotrienic acid（C20:3）	24.09±1.94^{a}	14.01±1.10^{c}	14.09±1.87^{c}	16.44±1.26^{bc}	17.68±0.16^{b}	19.19±1.43^{b}	16.52±0.81^{bc}
Erucic（C22:1）	24.65±1.81^{a}	17.24±1.13^{bc}	15.28±0.87^{c}	19.67±1.29^{b}	18.59±0.82^{bc}	18.17±1.33^{bc}	15.84±1.14^{c}
Cis 1,14,17-eicosotrienic acid（C20:3）	7.25±0.25^{a}	6.41±0.43^{a}	6.61±0.39^{a}	6.91±0.71^{a}	7.22±0.57^{a}	7.02±0.19^{a}	6.42±0.29^{a}
Twenty-three acid（C23:0）	10.29±0.52^{a}	1.28±0.07^{cd}	1.76±0.05^{bc}	1.27±0.06^{cd}	1.89±0.04^{b}	1.48±0.07^{bc}	0.73±0.04^{d}
Arachidonic acid（C20:4）	170.46±3.90^{a}	72.63±4.43^{d}	80.17±3.96^{d}	114.60±4.46^{b}	82.78±2.27^{d}	107.51±3.60^{b}	95.50±3.04^{c}
Docosadienoic acid（C22:2）	106.87±11.00^{a}	45.61±2.50^{c}	49.87±5.67^{c}	72.81±6.50^{b}	51.00±1.47^{c}	66.53±3.51^{b}	63.31±3.05^{b}
Lignoceric acid（C24:0）	5.68±0.37^{ab}	4.95±0.27^{c}	5.92±0.11^{a}	4.95±0.17^{c}	5.55±0.32^{abc}	5.43±0.10^{abc}	5.14±0.11^{bc}
Eicosapentaenoic acid（C20:5）	7.21±0.17^{a}	5.30±0.17^{d}	6.07±0.08^{c}	6.77±0.11^{b}	6.78±0.23^{b}	6.56±0.44^{b}	5.45±0.24^{d}
Tetracosenoic acid（C24:1）	6.14±0.12^{a}	5.07±0.16^{c}	5.57±0.38^{b}	5.18±0.16^{bc}	5.83±0.12^{ab}	5.51±0.15^{bc}	5.43±0.21^{bc}

DHA（C22:6）	30.33±3.20^{a}	14.10±0.91^{c}	14.67±0.17^{c}	18.01±1.66^{b}	15.05±0.84^{c}	20.89±1.18^{b}	11.33±0.41^{d}
SFA	1155.68±209.55^{a}	581.82±103.87^{de}	638.79±115.80^{d}	679.79±120.99^{cd}	731.91±131.72^{c}	907.90±163.22^{b}	791.49±141.97^{c}
MUFA	2060.01±577.20^{a}	1078.56±286.99^{e}	1292.68±348.82^{cd}	1189.32±320.48^{d}	1452.57±387.43^{c}	1740.74±472.15^{b}	1339.41±363.81^{c}

PUFA	2658.90±583.47[a]	1286.70±284.24[e]	1444.38±320.86[d]	1529.07±323.09[d]	1529.26±337.28[d]	1940.82±431.89[b]	1720.47±387.27[c]
TFFA	5874.59±463.37[a]	2947.07±227.96[e]	3375.85±265.29[d]	3398.19±257.81[d]	3713.74±287.37[c]	4589.47±358.84[b]	3851.36±301.93[c]

Note: Data with different letters (a, b, c, d, e) on the same line represent significant differences in the content of free fatty acids at different processing stages ($p<0.05$); In the table, SFA represents "saturated fatty acids", MUFA represents "monounsaturated fatty acids", PUFA represents "polyunsaturated fatty acids", and TFFA represents "total free fatty acids".

Table S7 Changes of soluble saccharides during the processing of passion fruit roasted chicken

Contents (mg/100g)	Different processing stages						
Name of soluble sugars	Raw chicken	After curing	Baking for 15 min	Baking for 30 min	Baking for 45 min	Baking for 60 min	Finished product
Trehalose (Tre)	0^{e}	8.96±0.39^{b}	7.74±0.24^{c}	7.70±0.24^{c}	5.73±0.16^{d}	9.67±0.33^{a}	8.18±0.67^{c}
Arabinose (Ara)	0^{d}	5.84±0.21^{b}	6.75±0.34^{a}	4.68±0.16^{c}	4.52±0.11^{c}	5.57±0.15^{b}	5.83±0.21^{b}
Glucose (Glc)	29.30±0.75^{e}	3942.72±85.15^{a}	3747.23±123.51^{b}	3219.62±79.44^{c}	2835.66±75.63^{d}	2979.77±82.94^{d}	3052.47±90.09^{d}
Fructose (Fru)	0^{f}	4633.73±70.54^{b}	4361.54±69.96^{c}	3778.80±84.94^{d}	3577.08±65.12^{e}	3460.91±70.36^{e}	3868.44±75.08^{d}
Ribose (Rib)	12.23±0.48^{e}	100.85±9.34^{a}	66.06±4.59^{d}	88.56±1.81^{b}	69.51±2.26^{cd}	74.28±2.80^{c}	76.86±2.90^{c}
Sucrose (Suc)	0^{d}	113.00±8.43^{a}	90.49±2.73^{b}	70.73±2.46^{c}	63.88±2.48^{c}	67.62±4.31^{c}	108.81±9.55^{a}
Lactose (Lac)	0^{e}	22.48±0.62^{a}	17.57±0.66^{b}	11.79±0.79^{d}	12.64±0.30^{d}	12.30±0.29^{d}	16.47±0.52^{c}
Stachyose (Sta)	0^{e}	206.86±8.06^{a}	186.12±5.56^{b}	194.40±8.00^{ab}	169.02±6.95^{c}	202.97±7.50^{a}	147.91±6.51^{d}
Maltose (Mal)	0^{f}	822.81±17.95^{b}	897.69±16.75^{a}	657.23±15.53^{d}	541.58±9.04^{e}	685.44±13.98^{c}	696.94±14.62^{c}
Total sugar contents	41.91±9.62^{d}	9857.25±1765.20^{a}	9381.19±1670.58^{a}	8033.51±1440.46^{b}	7279.62±1327.83^{c}	7498.53±1323.11^{bc}	7981.91±1431.28^{b}

Note: Data with different letters (a, b, c, d, e) on the same line represent significant differences in the content of soluble sugars at different processing stages ($p<0.05$).

Table S8 Changes of volatile flavor components of passion fruit roasted chicken during storage

Contents (ng/g)		Preservation time (d)					
Retention time (min)	Compounds	5	10	15	20	25	30
Aldehydes							
5.207	valeraldehyde	0^b	0^b	0^b	13.43 ± 2.36^a	0^b	0^b
7.657	hexanal	59.33 ± 4.01^b	86.42 ± 17.86^a	49.68 ± 3.76^c	29.24 ± 4.23^d	31.16 ± 2.87^d	0^e
10.172	Heptaldehyde	14.23 ± 2.63^b	44.23 ± 7.96^a	0^c	0^c	0^c	0^c
12.146	octanal	10.77 ± 4.23^b	47.82 ± 9.68^a	0^c	0^c	0^c	0^c
13.368	nonanal	37.02 ± 1.57^d	189.22 ± 22.67^a	68.36 ± 3.68^c	81.36 ± 5.62^b	71.55 ± 5.97^c	35.17 ± 3.65^d
17.205	Trans-2-dodecenal	0^b	44.64 ± 5.64^a	0^b	0^b	0^b	0^b
22.757	benzaldehyde	51.88 ± 5.55^d	106.48 ± 5.55^b	78.73 ± 8.21^c	121.91 ± 7.69^a	32.23 ± 2.35^e	28.47 ± 2.65^e
26.759	3-isopropylbenzaldehyde	0^b	0^b	0^b	10.07 ± 4.43^a	0^b	0^b
41.301	Myristic aldehyde	0^d	0^d	0^d	230.33 ± 16.87^a	189.69 ± 7.99^b	154.21 ± 7.98^c
41.530	Palmitic aldehyde	167.36 ± 14.32^b	212.47 ± 11.36^a	168.79 ± 5.54^b	0^c	0^c	0^c
45.931	(Z)-9-Octadecylenal	10.22 ± 3.87^a	0^b	0^b	0^b	0^b	0^b
	Subtotal	350.81 ± 49.83^c	731.28 ± 75.75^a	365.56 ± 54.48^c	486.34 ± 73.60^b	324.63 ± 57.91^c	217.85 ± 46.38^d
Ketones							
21.283	1,1,5-trimethyl-6-butenyl-4-cyclohexen-3-one	0^b	0^b	8.70 ± 2.38^a	7.28 ± 1.11^a	0^b	0^b
26.841	Geranyl acetone	0^c	7.31 ± 1.63^b	16.63 ± 6.70^a	0^c	0^c	0^c
29.403	ar-turmerone	0^c	0^c	69.06 ± 4.40^a	9.44 ± 3.01^b	0^c	0^c
29.691	2-methyl-6-(4-methylene-1-cyclo	0^b	0^b	49.30 ± 2.87^a	0^b	0^b	0^b

	hex-2-enyl) hept-2-en-4-one						
36.759	Aromatic ginger ketone	0^b	0^b	$27.22±4.56^a$	0^b	0^b	0^b
38.058	dihydro-β-ionone	$2.78±0.40^c$	$5.11±0.67^b$	$25.60±5.45^a$	$4.44±1.36^{bc}$	$3.56±0.36^c$	$5.93±0.31^b$
38.769	Ethyl Maltol	$280.57±16.96^a$	$189.74±20.07^b$	$163.04±15.99^b$	$122.37±10.67^c$	$79.88±4.79^d$	$53.66±6.32^e$
	Subtotal	$283.35±105.88^b$	$202.16±71.00^c$	$359.55±53.33^a$	$143.53±45.08^d$	$83.44±30.00^e$	$59.59±20.03^e$
alcohols							
3.856	ethanol	$353.17±10.57^d$	$264.02±18.21^e$	$488.47±27.56^c$	$283.44±10.84^e$	$749.33±23.66^a$	$566.46±18.78^b$
4.896	2,3-epoxyethane dimethanol	0^b	0^b	0^b	0^b	0^b	$8.52±1.50^a$
16.100	Linalool oxide	0^d	$8.99±2.86^a$	$7.65±2.32^b$	$6.46±2.20^c$	0^d	0^d
17.064	Linalool oxide	$16.14±6.63^d$	$33.09±7.73^b$	$45.72±7.87^a$	$25.28±2.14^c$	$18.22±4.32^d$	$16.84±3.01^d$
20.707	α-Terpineol	$16.73±4.58^d$	$37.70±4.20^a$	$27.65±4.21^b$	$28.24±3.32^b$	$20.88±1.69^c$	0^e
35.637	Isoeucalyptol	$3.15±0.56^c$	$9.35±1.23^b$	$14.93±1.66^a$	$13.87±1.52^a$	$9.74±1.58^b$	$10.89±1.60^b$
36.007	10,10-Dimethyl-2,6-dimethylene bicyclic [7.2.0] undecane carbon-5β-alcohol	0^e	$7.98±2.54^d$	$11.34±2.32^c$	$13.54±1.35^{ab}$	$14.78±0.54^a$	$15.78±6.35^a$
	Subtotal	$389.19±131.43^c$	$361.13±94.71^c$	$595.76±178.50^b$	$370.83±102.10^c$	$812.95±279.33^a$	$618.49±227.09^b$
Esters							
13.174	ethyl caproate	$19.32±4.21^c$	$26.95±2.57^b$	$37.72±1.54^a$	0^d	0^d	0^d
20.513	hexyl	$4.73±0.58^d$	$10.72±1.57^c$	$16.59±2.87^a$	$13.90±2.22^b$	$9.14±0.74^c$	$9.78±1.04^c$

	butyrate						
20.701	Ethyl octanoate	52.31±6.23^{a}	47.68±6.88^{ab}	38.39±5.41^{c}	0^{d}	0^{d}	0^{d}
21.283	1-Methylbutyrate hexyl ester	29.34±3.21^{a}	22.27±4.63^{b}	18.10±1.69^{c}	14.60±2.38^{d}	13.87±0.98^{d}	11.36±2.79^{d}
27.423	Hexyl Hexanoate	15.39±1.23^{c}	43.44±2.87^{b}	70.84±4.21^{a}	37.14±3.98^{bc}	28.25±3.98^{d}	43.36±3.21^{b}
27.728	ethyl decanoate	10.45±1.85^{c}	25.09±5.56^{b}	34.26±6.02^{a}	24.54±1.04^{b}	21.14±3.65^{b}	25.78±3.68^{b}
27.958	3-Heptanylhexanoate	18.61±4.36^{d}	44.47±4.96^{b}	109.41±9.98^{a}	38.90±2.87^{b}	30.60±2.05^{c}	41.40±2.88^{b}
28.768	Ethyl octadecanoate	0^{b}	0^{b}	0^{b}	0^{b}	4.31±1.07^{a}	0^{b}
33.986	Ethyl laurate	10.99±1.87^{c}	13.01±1.99^{b}	27.25±5.47^{a}	12.06±0.69^{b}	11.12±2.15^{c}	12.73±1.04^{b}
40.596	ethyl myristate	3.84±0.85^{d}	5.80±0.71^{c}	6.29±0.66^{b}	6.54±0.44^{b}	6.97±0.99^{b}	10.86±0.39^{a}
44.174	methyl palmitate	3.88±2.11^{b}	4.59±0.20^{b}	15.95±1.21^{a}	0^{c}	0^{c}	0^{c}
45.802	Ethyl Palmitate	21.05±1.21^{c}	17.99±1.05^{d}	18.23±0.77^{d}	18.17±2.98^{d}	30.67±2.89^{b}	37.70±3.77^{a}
49.439	ethyl linoleate	3.98±0.54^{a}	0^{b}	0^{b}	0^{b}	0^{b}	0^{b}
	Subtotal	193.89±14.15^{c}	262.01±16.76^{b}	393.03±30.48^{a}	165.85±13.86^{cd}	156.07±11.95^{d}	192.97±16.63^{c}
Hydrocarbons							
5.813	heptane	0^{b}	0^{b}	0^{b}	0^{b}	68.41±1.58^{a}	0^{b}
12.616	3-carene	20.35±5.78^{e}	30.44±5.45^{cd}	79.51±2.41^{a}	53.65±4.15^{b}	39.95±5.54^{c}	35.71±2.34^{c}
12.928	4-methyl-1-(1-methylethyl) -bicyclic [3.1.0] -2-hexene	0^{b}	0^{b}	0^{b}	0^{b}	0^{b}	10.13±1.97^{a}
13.768	Limonene	27.76±4.68^{c}	13.08±1.58^{e}	34.15±2.69^{b}	16.20±0.24^{d}	70.22±4.54^{a}	16.94±2.71^{d}
13.915	Cyclized fennel	0^{b}	0^{b}	0^{b}	38.34±2.87^{a}	0^{b}	0^{b}
14.297	α-Terpinene	0^{d}	0^{d}	19.94±2.65^{a}	11.32±0.41^{b}	7.30±1.01^{c}	7.59±1.02^{c}

14.461	α-pinene	18.05±6.33^{b}	22.26±3.78^{a}	23.34±3.11^{a}	25.94±2.31^{a}	15.45±0.81^{b}	0^{c}
16.054	4,7-Methyleneoctahydroindene	0^{c}	0^{c}	0^{c}	0^{c}	145.02±1.57^{a}	32.54±3.65^{b}
17.305	ocimene	0^{b}	0^{b}	0^{b}	0^{b}	15.55±1.30^{a}	0^{b}
23.903	4,7-Dimethylundecane	0^{b}	0^{b}	0^{b}	8.41±2.11^{a}	0^{b}	0^{b}
26.048	4-isopropyltoluene	5.62±1.20^{b}	5.87±0.68^{b}	6.81±1.01^{a}	3.82±0.31^{cd}	4.91±0.99^{bc}	0^{e}
27.611	Tetradecane	3.22±1.11^{d}	5.56±0.21^{b}	7.14±0.11^{a}	4.59±2.31^{c}	0^{e}	0^{e}
28.980	β-caryophyllene	65.51±4.01^{e}	275.42±17.89^{d}	459.91±32.10^{a}	366.71±17.88^{b}	301.26±20.40^{c}	375.67±18.88^{b}
29.473	Guaiacol	4.32±1.32^{e}	5.48±0.41^{de}	12.44±1.99^{a}	6.62±0.69^{c}	6.30±1.30^{cd}	8.46±0.71^{b}
29.896	2,6,10-trimethyltriadecane	20.37±3.21^{b}	21.45±1.58^{ab}	23.59±2.45^{a}	0^{c}	0^{c}	0^{c}
30.090	α-caryophyllene	6.42±1.55^{e}	24.98±2.96^{d}	51.95±2.77^{a}	32.46±2.10^{bc}	28.06±1.20^{cd}	39.79±1.21^{b}
30.789	α-Curcumene	0^{b}	0^{b}	27.14±3.68^{a}	0^{b}	0^{b}	0^{b}
30.925	1-(1,5-dimethyl-4-hexenyl)-4-methylbenzene	0^{b}	0^{b}	0^{b}	6.39±0.24^{a}	0^{b}	0^{b}
31.148	β-Selinene	42.97±7.74^{c}	46.61±2.57^{c}	52.36±5.41^{b}	61.51±7.77^{a}	59.28±4.42^{a}	0^{d}
31.401	α-Selinene	17.61±6.28^{e}	28.37±6.87^{d}	114.85±10.77^{a}	36.26±2.35^{c}	38.40±0.98^{c}	45.17±3.97^{b}
31.595	β-bisabolene	8.79±2.14^{e}	27.01±3.88^{b}	54.31±2.99^{a}	16.88±1.36^{d}	16.06±2.39^{d}	19.40±1.54^{c}
32.094	β-Sedumene sesquihydrate	0^{b}	0^{b}	70.53±6.40^{a}	0^{b}	0^{b}	0^{b}
32.147	1,2,3,5,6,8a-Hexahydro-4,7-dimethyl-1-isopropylnaphthalene	0^{e}	8.70±0.87^{cd}	9.78±1.96^{c}	14.09±0.98^{a}	13.01±2.11^{ab}	13.12±0.69^{ab}

32.781	2,2-dimethyl-3-octene	10.94±1.35^{a}	0^{b}	0^{b}	0^{b}	0^{b}	0^{b}
34.215	Caryophyllene oxide	24.72±1.44^{e}	41.86±2.64^{d}	87.57±5.58^{a}	57.23±2.37^{c}	62.33±1.87^{b}	40.76±2.23^{d}
34.479	3-(1-methylethene) cyclooctene	0^{b}	0^{b}	0^{b}	0^{b}	36.35±2.31^{a}	0^{b}
34.726	β-elemene	0^{b}	0^{b}	37.57±2.99^{a}	0^{b}	0^{b}	0^{b}
35.795	7.9-Dimethylhexadecane	0^{b}	0^{b}	21.54±6.32^{a}	0^{b}	0^{b}	0^{b}
38.175	1,13-Tetradecadiene	0^{b}	12.54±0.54^{a}	0^{b}	0^{b}	0^{b}	0^{b}
	Subtotal	276.65±15.58^{e}	569.63±51.11^{d}	1194.43±86.29^{a}	760.42±68.15^{c}	927.86±61.33^{b}	645.28±69.55^{d}
Others							
13.762	2-Pentylfuran	7.56±2.11^{a}	0^{b}	0^{b}	0^{b}	0^{b}	0^{b}
24.438	Anethole	27.34±3.74^{b}	23.29±2.21^{b}	31.31±2.41^{a}	15.97±3.33^{c}	0^{d}	0^{d}
31.712	2,6-Di-tert-butyl-p-cresol	6.04±1.69^{e}	14.37±1.26^{ab}	16.43±3.08^{a}	11.86±1.21^{c}	6.79±0.67^{e}	9.98±2.22^{cd}
	Subtotal	40.94±11.88^{c}	37.66±11.75^{b}	47.74±15.66^{a}	27.83±8.29^{c}	6.79±0.67^{d}	9.98±2.22^{d}
	Total	1534.83±56.69^{e}	2163.87±59.14^{b}	2956.07±83.09^{a}	1954.80±62.93^{c}	2311.74±99.50^{b}	1744.16±82.46^{d}

Note: Data with different letters (a, b, c, d, e) in the same row represent significant differences in the content of volatile flavor components for different storage days ($p<0.05$).

Table S9 Comparison of volatile flavor compounds between passion fruit roasted chicken and other pre-made chicken products

No	Compounds	Passion Fruit Roasted Chicken (Thi	Fuliji Roasted Chicken[2,6]	Daokou Roasted Chicken[1,3]	Professor Huang Roasted Chicken[7]	Wang Si Roasted Chicken[7]	Texas Braised Chicken[1,4,5]	Jingning Braised Chicken[7]	Ziyan Baiwei Chicken[7]	Ma Yuxing Roasted Chicken[7]

		s<br>stud<br>y)								
	**aldehydes**									
1	isovaleraldehyde	+	–	–	–	+	–	–	+	+
2	valeraldehyde	+	+	+	+	+	+	+	+	–
3	nonanal	+	+	+	+	+	+	+	+	+
4	benzaldehyde	+	+	+	+	+	+	+	+	+
5	hexanal	–	+	+	+	+	+	+	+	+
6	heptaldehyde	–	+	+	+	–	+	–	+	+
7	furfural	–	–	–	–	–	+	–	–	–
8	acetal	–	+	–	+	+	–	–	–	+
9	octanal	–	–	–	+	–	–	–	–	–
10	decanal	–	–	–	+	–	+	–	+	+
11	3-Furfural	+	–	–	–	–	–	–	–	–
	**furans**									
1	2-Pentylfuran	+	–	+	+	–	+	+	+	+
	**ketones**									
1	2-Nonanone	–	–	–	+	–	+	–	–	–
2	benzylacetone	–	+	–	–	–	–	–	–	–
3	2,5-Octadione	–	–	+	+	–	–	–	–	–
4	camphor	–	+	+	–	–	+	+	+	+
5	fenchone	–	–	–	–	–	+	+	–	–
6	piperitone	–	+	–	–	–	+	–	–	+
7	5-methyl-2-hexanone	+	–	–	–	–	–	–	–	–
8	3-hydroxy-2-butanone	+	–	–	–	–	–	–	–	–
9	2-furanylacetone	+	–	–	–	–	–	–	–	–
	**alcohols**									
1	2-methylbutanol	+	–	–	–	–	–	–	–	–
2	2-Heptanol	+	–	–	–	–	–	–	–	–
3	Hexanol	+	–	–	–	–	–	–	–	–
4	(S) - Linalool oxide	+	–	–	–	–	–	–	–	–
5	L-Linalool	+	–	–	–	–	–	–	–	–
6	Terpineol	+	–	–	–	–	–	–	–	–
7	Phenylethanol	+	–	–	–	–	–	–	–	–
8	isoamyl alcohol	–	–	–	–	+	–	–	–	–
9	furfuryl alcohol	–	–	–	+	–	+	–	–	–
10	1-octen-3-ol	–	–	+	+	+	–	+	–	–
11	Eucalyptus oleanol	–	+	+	–	+	+	+	+	+
12	2,2-dimethyloctanol	–	–	–	–	–	–	–	–	+
13	Cis-4- (isopropyl) -1-methylcyclohex-2-en-1-ol	–	–	–	–	+	–	–	–	–
14	linalool	–	+	+	–	+	+	+	–	+

15	2-Butyl octanol	--	--	+	--	--	--	--	--	--
16	2-tsol	--	--	--	--	+	--	+	--	--
17	terpinen-4-ol	--	+	+	+	+	+	+	+	+
18	α-Terpineol	--	+	+	--	+	+	+	+	+
19	2-Butyl-1-octanol	--	--	--	--	--	--	+	--	--
20	2-hexyl octanol	--	--	--	--	--	--	+	--	--
	**Acids and esters**									
1	acetic acid	+	--	--	--	+	--	--	--	--
2	ethyl acetate	+	--	--	--	--	--	--	--	--
3	Ethyl Butyrate	+	--	--	--	--	--	--	--	--
4	ethyl lactate	+	--	--	--	--	--	--	--	--
5	1-Methylhexyl ester	+	--	--	--	--	--	--	--	--
6	Hexyl butyrate	+	--	--	--	--	--	--	--	--
7	Ethyl octanoate	+	--	--	--	--	--	--	--	--
8	butyric acid	--	--	--	--	+	--	--	--	--
9	ethyl 3-hydroxybutyrate	+	--	--	--	--	--	--	--	--
10	butyl acetate	--	--	--	--	--	--	--	+	+
11	Eugenyl acetate	--	--	--	--	--	--	--	+	--
12	Bornyl acetate	--	--	+	--	--	+	--	+	--
13	Acetic acid fennel ester	--	--	+	--	--	--	--	--	--
14	ethyl caproate	+	--	--	+	--	--	--	--	--
15	N-Amyl nitrate	--	--	+	--	--	--	--	--	+
16	isobutyl isobutyrate	--	+	--	--	--	--	--	--	--
17	Butyl isobutyrate	--	--	--	--	--	--	--	+	--
	**Hydrocarbons**									
1	3-carene	+	+	+	--	--	--	--	+	--
2	γ-Terpinene	+	+	+	+	+	+	+	+	+
3	α-phellandrene	+	+	+	--	+	--	--	--	--
4	β-myrcene	+	--	--	--	--	--	--	--	--
5	(+) - limonene	+	--	--	--	--	--	--	--	--
6	ocimene	--	+	--	--	--	+	--	--	--
7	styrene	+	--	--	--	--	--	--	--	--
8	Guaiacol	+	--	--	--	--	--	--	--	--
9	β-caryophyllene	+	--	--	--	--	--	--	--	--
10	Z. Z, Z-1,5,9,9-tetramethyl-1,4,7-cycloundecane triene	+	--	--	--	--	--	--	--	--
11	ethylbenzene	+	--	--	--	--	--	--	--	--
12	Paraxylene	+	--	+	+	+	+	+	--	+
13	1-Pentene	+	--	--	--	--	--	--	--	--
14	Anisin	+	--	--	--	--	--	--	--	--

15	benzene	–	+	+	+	–	+	+	–	+
16	methylbenzene	+	+	+	+	+	+	+	+	+
17	octane	–	–	–	–	–	–	–	–	+
18	ethyl benzene	–	+	+	+	+	+	+	+	+
19	m-xylene	–	–	–	+	–	–	–	+	+
20	O-Xylene	–	+	+	+	–	–	–	+	–
21	2-pinene	–	+	+	–	+	+	–	+	+
22	camphene	–	+	+	–	+	+	–	+	–
23	β-phellandrene	–	–	–	–	+	–	–	–	–
24	β-pinene	–	+	+	–	+	+	–	+	+
25	2,2,4,6,6-pentamethyl heptane	–	–	–	–	–	–	+	–	–
26	myrcene	–	+	–	–	+	–	–	–	–
27	decane	–	+	+	+	–	+	+	+	+
28	2-Carene	–	–	–	–	+	–	–	–	–
29	Terpinene	–	+	+	+	–	+	–	–	+
30	O-isopropyltoluene	–	+	+	–	–	+	–	–	–
31	M-isopropyltoluene	–	–	–	+	–	–	–	–	–
32	4-isopropyltoluene	–	–	–	–	+	–	–	+	+
33	Limonene	–	+	+	+	+	+	+	+	+
34	(E)-β-ocimene	–	+	–	–	–	+	–	–	–
35	ocimene	+	+	–	–	–	+	–	–	–
36	γ-Terpinene	–	+	+	+	+	+	+	+	+
37	terpinolene	–	+	+	–	–	–	–	–	–
38	1-methyl-4-(1-methylvinyl) benzene	–	–	–	–	+	–	–	–	–
39	4-Isopropenyltoluene	–	–	–	–	–	+	–	–	–
40	Undecane	–	–	–	+	–	+	–	–	–
41	dodecane	–	–	–	+	–	+	+	+	+
42	Estragol	–	+	+	–	+	–	+	+	–
43	Anethole	+	+	+	+	+	+	+	+	+
44	tridecane	–	–	–	+	–	+	–	–	–
45	α-pinene	–	+	+	+	+	+	–	+	–
46	tetradecane	–	+	+	+	–	+	+	+	+
47	α-selinene	–	–	–	–	+	–	+	–	–
48	Longifolene	–	+	–	+	–	–	–	–	–
49	caryophyllene	–	+	–	+	+	–	–	+	+
50	2,6-Dimethyl-6-(4-methyl-3-pentenyl) bicyclic [3.1.11] hept-2-ene	–	–	–	–	+	–	–	–	–
51	α-Lycaene	–	+	–	–	+	–	–	–	–
52	cetane	–	–	+	–	–	+	–	+	+

53	heptadecane	--	--	+	--	--	+	--	+	+
54	Octadecane	--	+	--	+	--	+	--	+	+
55	nonadecane	--	+	--	+	--	--	--	--	--
56	Eicosane	--	--	--	+	--	--	--	--	--
	**Ethers**									
1	dimethyl ether	+	--	--	--	--	--	--	--	--
2	Methyl disulfide	--	--	--	+	--	--	--	--	--
	**Phenols**									
1	Ethyl Maltol	+	--	--	--	--	--	--	--	--
2	3,5-dimethylphenol	--	--	--	--	--	+	--	--	--
3	Eugenol	--	+	--	--	+	+	--	+	+
	**pyrazines**									
1	2,6-Dimethylpyrazine	--	--	--	--	--	+	--	+	--
2	3-ethyl-2,5-methylpyrazine	--	--	--	--	--	+	--	--	--

Table S10 Abbreviation and full name

Abbreviation	Full name
BFAA	Bitter free amino acids
GC-MS	Gas chromatography-mass spectrometer
HS-SPME	Headspace-solid phase micro extraction
LRI	Linear retention index
MUFA	Monounsaturated fatty acid
OAV	Odor activity value
PES	*Passiflora edulis* Sims
PUFA	Polyunsaturated fatty acid
SFA	Saturated fatty acid
SFAA	Sweet free amino acids
TFAA	Tasteless free amino acids
UFAA	Umami free amino acids
